# Dormancy-associated MADS-box genes and microRNAs jointly control dormancy transition in pear (*Pyrus pyrifolia* white pear group) flower bud

**DOI:** 10.1093/jxb/erv454

**Published:** 2015-10-14

**Authors:** Qingfeng Niu, Jianzhao Li, Danying Cai, Minjie Qian, Huimin Jia, Songling Bai, Sayed Hussain, Guoqin Liu, Yuanwen Teng, Xiaoyan Zheng

**Affiliations:** ^1^Department of Horticulture, Zhejiang University, Hangzhou, Zhejiang 310058, China; ^2^The Key Laboratory of Horticultural Plant Growth, Development and Quality Improvement, the Ministry of Agriculture of China, Hangzhou, Zhejiang 310058, China; ^3^Zhejiang Provincial Key Laboratory of Integrative Biology and Utilization of Horticultural Plants, Hangzhou, Zhejiang 310058, China; ^4^Institute of Horticulture, Zhejiang Academy of Agricultural Sciences, Hangzhou, Zhejiang Province 310021, China; ^5^College of Agriculture, Guizhou University, Guiyang, Guizhou Province 550025, China; ^6^Institute of Horticulture and Landscape, College of Ecology, Lishui University, Lishui, Zhejiang 323000, China

**Keywords:** Dormancy, microRNA, MIKC^C^-type MADS-box genes, *Pp*CBF, *PpFT2*, transient assays, yeast one-hybrid.

## Abstract

Bud dormancy in perennial plants is indispensable to survival over winter and to regrowth and development in the following year. However, the molecular pathways of endo-dormancy induction, maintenance, and release are still unclear, especially in fruit crops. To identify genes with roles in regulating endo-dormancy, 30 MIKC^C^-type MADS-box genes were identified in the pear genome and characterized. The 30 genes were analysed to determine their phylogenetic relationships with homologous genes, genome locations, gene structure, tissue-specific transcript profiles, and transcriptional patterns during flower bud dormancy in ‘Suli’ pear (*Pyrus pyrifolia* white pear group). The roles in regulating bud dormancy varied among the *MIKC* gene family members. Yeast one-hybrid and transient assays showed that *Pp*CBF enhanced *PpDAM1* and *PpDAM3* transcriptional activity during the induction of dormancy, probably by binding to the C-repeat/DRE binding site, while DAM proteins inhibited the transcriptional activity of *PpFT2* during dormancy release. In the small RNA-seq analysis, 185 conserved, 24 less-conserved, and 32 pear-specific miRNAs with distinct expression patterns during bud dormancy were identified. Joint analyses of miRNAs and *MIKC* genes together with degradome data showed that miR6390 targeted *PpDAM* transcripts and degraded them to release *PpFT2*. Our data show that cross-talk among *PpCBF*, *PpDAM*, *PpFT2*, and miR6390 played important roles in regulating endo-dormancy. A model for the molecular mechanism of dormancy transition is proposed: short-term chilling in autumn activates the accumulation of CBF, which directly promotes *DAM* expression; DAM subsequently inhibits *FT* expression to induce endo-dormancy, and miR6390 degrades *DAM* genes to release endo-dormancy.

## Highlight

Short-term chilling in autumn activates the accumulation of CBF, which directly promotes *DAM* expression. DAMs subsequently inhibit *FT2* expression to induce endo-dormancy; miR6390 might degrade *DAM* genes to release endo-dormancy.

## Introduction

An important characteristic of temperate perennial plants is their ability to maintain a dormant state. In this state, the meristem is rendered insensitive to growth-promoting signals for some time before dormancy is released and the plants resume growth (Lang, 1996; Horvath *et al.*, 2003; Rohde and Bhalerao, 2007). During the perennial plant life cycle, buds transit through the various stages of dormancy (para-, endo-, and eco-dormancy, as defined by Lang *et al.*, 1987). Dormancy regulation in buds is a complex process that is necessary for plant development, productivity, adaptability, survival, and distribution (Chuine and Beaubien, 2001). Knowledge about mechanisms regulating dormancy induction, maintenance, and release may provide the basis for solving critical problems in agriculture (Anderson *et al.*, 2010), especially irregular blooming and prolonged flowering periods in deciduous fruit trees cultivated in temperate regions. Recent genomics- and transcriptomics-based studies have provided insights into some of the basic aspects of the molecular mechanisms of dormancy regulation (Horvath *et al.*, 2008; Liu *et al.*, 2012; Bai *et al.*, 2013).

Some MADS-box genes, such as the *Dormancy-associated MADS-box* genes (*DAM*s), have been identified as the internal factors controlling endo-dormancy in perennial species (Horvath *et al.*, 2008, 2010; Li *et al.*, 2009; Sasaki *et al.*, 2011). *DAM* genes have been implicated in regulating bud dormancy in raspberry (Mazzitelli *et al.*, 2007), leafy spurge (Horvath *et al.*, 2010), potato (Campbell *et al.*, 2008), apricot (Sasaki *et al.*, 2011), peach (Li *et al.*, 2009; Leida *et al.*, 2010, 2012), apple (Mimida *et al.*, 2015), and pear (Liu *et al.*, 2012). *DAM* genes, which are closely related to *SHORT VEGETATIVE PHASE* (*SVP*) MADS-box genes and are located at the *Evergrowing* (*EVG*) locus, have recently been cloned from a non-dormant *evg* mutant in peach (Bielenberg *et al.*, 2008). A seasonal expression analysis of these *DAM* genes showed that they were up-regulated during endo-dormancy induction and down-regulated during endo-dormancy release (Bielenberg *et al.*, 2008; Jimenez *et al.*, 2009; Li *et al.*, 2009; Sasaki *et al.*, 2011; Wu *et al.*, 2012). Sequencing of the *EVG* locus from wild-type and mutant lines revealed a series of *MIKC* ^*C*^ *-type MADS-box* genes (*MIKC* genes) that were missing from the mutant lines (Bielenberg *et al.*, 2008).

*MIKC* orthologues are suspected to play a role in dormancy regulation, and have been identified in several perennial plants (Hoenicka *et al.*, 2008; Jimenez *et al.*, 2009; Horvath *et al.*, 2010; Wu *et al.*, 2012). For instance, *AGL24*, a member of the *MIKC* gene family, was shown to be up-regulated in *Arabidopsis* during cold temperature (Lee *et al.*, 2007). Analyses of available transcriptome data have indicated that *MIKC* genes are regulated by environmental conditions that affect bud dormancy in perennial species (Horvath *et al.*, 2008; Liu *et al.*, 2012). *MIKC* genes may also play roles in dormancy maintenance and release through regulating the *FLOWERING LOCUS T* (FT) gene (Horvath *et al.*, 2008, 2010; Sasaki *et al.*, 2011). Horvath *et al.* (2010) showed that *FT* expression was down-regulated in transgenic *Arabidopsis* lines overexpressing leafy spurge *DAM1*, and these transgenic *Arabidopsis* lines also showed delayed flowering compared with that in the wild type. In *Populus* (poplar), *FT* encodes a major long-distance signal that is hyperinduced by chilling and plays a role in regulating dormancy release (Hsu *et al.*, 2011; Rinne *et al.*, 2011).

Plants have evolved a suite of mechanisms to adapt to harsh environments and survive during the cooler seasons (Anderson *et al.*, 2010). Endo-dormancy induction, maintenance, and release in many perennial plants, including pear, depend mainly on a sufficient accumulation of the chilling temperature (Heide and Prestrud, 2005). C-repeat binding factors (CBFs) are well-characterized transcription factors involved in the cold temperature response pathway (Kendall *et al.*, 2011; Wisniewski *et al.*, 2011). The transcript levels of *CBF*s increase rapidly in response to cold temperature. In several studies, overexpression of *CBF*s enhanced freezing tolerance in the absence of cold acclimation as a result of the up-regulated expression of a series of genes involved in metabolic and physiological changes that aid freezing resistance (Gilmour *et al.*, 2000; Wisniewski *et al.*, 2011). A notable feature of both *CBF* overexpression and low temperature is that both cause marked growth retardation through the promotion of GA catabolism, supporting a model in which CBFs act in parallel with a cold temperature signalling pathway to regulate dormancy (Kendall *et al.*, 2011). Constitutive overexpression of *CBF1* in apple resulted in short-day-induced dormancy and a 4–6 °C increase in freezing tolerance (Wisniewski *et al.*, 2011). CBF-binding sites have been found in *DAM* promoters in leafy spurge (Horvath, 2009; Horvath *et al.*, 2010). Thus, among perennial species, the presence of CBF-binding sites in *DAM* promoters might explain why cold is the primary signal inducing endo-dormancy.

Plants reprogramme their gene expression profiles to cope with cold temperatures that adversely affect normal growth. A previous study showed that plant genomes are particularly vulnerable to epigenetic changes induced by environmental factors (Turner, 2000). The expressions of some microRNAs (miRNAs) change during cold acclimation. For instance, miR156 and miR172 were reported to be involved in regulating the timing of sensitivity of the vernalization response in *Cardamine flexuosa*, while age and vernalization pathways were shown co-ordinately to regulate flowering by modulating the expression of *CfSOC1*, an *MIKC* gene that promotes flowering (Zhou *et al.*, 2013). In *Arabidopsis*, miR156, which targets *SQUAMOSA PROMOTER BINDING–LIKE* (*SPL*) transcription factors, was shown to regulate age-dependent developmental transitions (Wang *et al.*, 2009; Wu *et al.*, 2009). However, little is known about the role of miRNAs in regulating dormancy in perennial plants. Recently, RNA-seq (short-read high-throughput sequencing) has become a popular and powerful tool for sequencing miRNAs and quantifying their expression. High-throughput degradome sequencing, a method known as parallel analysis of RNA ends, has been successfully established and adapted to validate miRNA splicing targets in various plants (German *et al.*, 2008). This method provides a new and efficient strategy to confirm predicted miRNA targets on a large scale in plants. However, until now, this technology has not been used to unravel the molecular components that govern the transitions into and out of dormancy, particularly at the epigenetic level (Horvath, 2009; Anderson *et al.*, 2010).

Pears (*Pyrus* spp.) are among the world’s most important perennial deciduous fruit trees. These species respond to chilling temperature to transit from growth to dormancy during their annual growth cycles. Most studies on pear dormancy have been at the physiological level, focusing on respiration (Bi *et al.*, 2011), carbohydrate (Zimmerman and Faust, 1969) and protein metabolism (Tamura *et al.*, 1998), and chilling requirements (Rufato *et al.*, 2011). Two *DAM* genes have been isolated from *Pyrus pyrifolia* and their expression patterns during the endo-dormancy transition phases have been reported (Ubi *et al.*, 2010). Two independent transcriptomic analyses of pear buds have provided valuable resources for the identification of pear genes involved in bud dormancy (Liu *et al.*, 2012; Bai *et al.*, 2013). Both of these studies found that down-regulation of *DAM* genes was concomitant with endo-dormancy release, consistent with the results of previous studies on peach (Li *et al.*, 2009; Leida *et al.*, 2012) and Japanese apricot (Sasaki *et al.*, 2011). However, these results are still insufficient to elucidate the molecular regulation mechanism of endo-dormancy induction, maintenance, and release. Furthermore, with global warming, many deciduous fruit trees (including pear) growing in warm areas have shown irregular phenologies resulting from inadequate winter chilling, which is unfavourable for sustainable fruit production (Luedeling *et al.*, 2011). Therefore, understanding the molecular regulation mechanisms of dormancy transition in fruit trees will be useful for developing strategies to breed cultivars with lower chilling requirements and to develop agronomic measures to cope with insufficient chilling.

The pear genome sequence was analysed here (Wu *et al.*, 2013) and genome-wide pear *MIKC* genes were identified and characterized. The transcript profiles of these genes were analysed in five different organs/tissues and their transcriptional patterns in buds at different dormancy stages. The results provide a framework for studying the biological function of *MIKC* genes during bud dormancy. As part of a long-term goal to elucidate the role of miRNAs in bud dormancy in pear, RNA-seq, degradome sequencing, and computational and molecular analyses were used comprehensively to identify conserved and pear-specific miRNAs and their targets, and to determine their expression profiles in flower buds during dormancy. A miRNA-mediated regulatory network that could modulate the genes involved in bud dormancy was also delineated. This network has not been reported in other species. In addition, a genome-wide identification and analysis of miRNAs that might target *MIKC* genes to regulate dormancy transition was performed. The yeast one-hybrid assay and transient assays were used to validate the interaction between *PpCBF* and *PpDAM*, and between *PpDAM* and *PpFT2*. In this study, a model of the molecular regulation network affecting dormancy transition in pear flower buds was established. Together, these results contribute to a better understanding of the regulation of bud dormancy in perennial plants.

## Materials and methods

### Plant materials and RNA isolation

Ten-year-old ‘Suli’ pear trees (*Pyrus pyrifolia* white pear group) grafted on to *P. betulaefolia* Bunge rootstocks cultivated in the Dangshan Germplasm Resources Center (Dangshan County, Anhui Province, China) were used in this study. The trees used in these experiments were not pruned or chemically treated. All bud samples were collected from the same trees at each dormancy stage, frozen in liquid nitrogen, and stored at –80 °C before RNA extraction. Transcripts and expression analyses were performed on lateral flower buds collected on 15 November, 15 December, 8 January, 15 January, 25 January, 15 February, and 8 March (from November 2010 to March 2011). Various organs were also collected for tissue- (organ-) specific gene expression analyses. Roots of *P. betulaefolia* rootstocks and leaves of ‘Suli’ pear were collected on 15 September 2010, and lateral flower buds and stems of ‘Suli’ pear were collected on 15 December 2010. All materials were collected for three biological replicates.

Total RNA was extracted using pBiozol Total RNA Extraction Reagent (BioFlux, Hangzhou, China) according to the manufacturer’s instructions, and genomic DNA was removed by DNase I (Takara, Kyoto, Japan). The RNA solutions were then subjected to extra chloroform extraction and ethanol precipitation ethanol at –20 °C overnight.

### Dormancy status of lateral flower buds

The dormancy status of lateral flower buds on the seven collection dates from 2010 to 2011 was estimated as described previously (Liu *et al.*, 2012). To measure the percentage bud break, 12 shoots from the current season’s growth, approximately 60-cm long and bearing apical buds, and 10–12 lateral flower buds were collected. The shoots were placed in water in 500ml vials in a phytotron and kept under a day/night temperature of 25±1/18±1 °C, with a 12h photoperiod of white light (320 μmol photons m^–2^ s^–1^) and 75% humidity. The water in the vials was changed and the basal ends of the shoots were cut every 2–3 d. After 21 d, the dormancy status was evaluated by determining the percentage bud break; the beginning of bud break was defined as green leaf tips enclosing visible flowers. Lateral flower buds of shoots with bud break percentages of less than 50% were considered to have remained in the endo-dormant stage (Lang *et al.*, 1987).

### Small RNA library construction and sequencing

Total RNA was isolated from lateral flower buds collected on 15 November (20101115A), 15 December (20101215A), 15 January (20110115A), and 15 February (20110215A). Four independent small RNA libraries (20101115A, 20101215A, 20110115A, and 20110215A) were constructed and sequenced using the Illumina HiSeq™ 2000 platform (Illumina, San Diego, CA, USA). The 49-nucleotide-long sequence tags from the Illumina sequencing were filtered to remove low-quality tags and 5′ adaptor contaminants to obtain credible clean tags. The clean tags were searched against the GenBank and Rfam 10.0 databases (Kozomara and Griffiths-Jones, 2014) to identify and remove rRNAs, scRNAs, snoRNAs, snRNAs, and tRNAs. The remaining sRNA tags were aligned to the mRNA sequences to identify and remove any degraded mRNA fragments (http://peargenome.njau.edu.cn:8004/default.asp?d=4&m=2) (Wu *et al.*, 2013). Only sRNA tags that formed good stem-loop structures and had a miRNA/miRNA* pair were considered as potential miRNAs. The criteria used to identify the candidate miRNAs were described previously by Niu *et al.* (2013). The potential miRNAs were then mapped to the pear genome sequence (http://peargenome.njau.edu.cn:8004/default.asp?d=4&m=2) (Wu *et al.*, 2013) by SOAP 2.20 (http://soap.genomics.org.cn/soapsplice.html), and their distribution on the genome and expression were analysed.

### Genome-wide identification of pear miRNAs and their expression during bud dormancy

The high-throughput sequencing abundance profile analysis was based on the numbers of reads in each library during bud dormancy. The expression levels of the miRNAs in the four libraries were transformed to transcripts per million normalized values as follows: normalized expression=actual miRNA count/(total count of clean reads×1 000 000).

The *P*-value used to determine the significance of differences in miRNA levels among the four libraries was calculated using previously established methods (Ruby *et al.*, 2006). All calculations were performed on the BGI Bio-Cloud Computing platform (http://cloud.genomics.org.cn). MiRNA tags per million values of less than 1 were removed from the libraries.

A target *t* test was performed among the sample groups. The *t* values were calculated for each miRNA and *P* values were computed from the theoretical *t* distribution (Man *et al.*, 2000). Only miRNAs with *P* <0.01 were selected for the cluster analysis. The clustering plot was generated using TIGR MeV software (http://www.tm4.org/) (Eisen *et al.*, 1998).

### Quantitative real-time PCR validation

First-strand cDNA was synthesized from 1 μg DNA-free RNA using the SYBR^®^ PrimeScript miRNA RT-PCR Kit (Takara, Kyoto, Japan) according to the manufacturer’s instructions. The forward miRNA primers for real-time PCR were designed from the full pear miRNA sequences, and the reverse primer was the universal reverse primer for miRNAs. The primer sequences are listed in Supplementary Table S8 in Supplementary File 3 at *JXB* online. The reactions were performed on a LightCycler 1.5 instrument (Roche, Basel, Switzerland) according to the manufacturer’s instructions. The specificity of the qRT-PCR primers was confirmed by melting curves and sequencing of the qRT-PCR products. Each reaction was repeated three times. The miRNA transcript levels were quantified using the comparative 2^–ΔΔCt^ method. 5S rRNAs was used as an internal control (Design, 2005; Wu *et al.*, 2014). The data were analysed using the Data Processing System (version 7.05; Zhejiang University, Hangzhou, China).

### Target identification by degradome sequencing

Equal amounts of RNA from the four independent lateral flower bud libraries were pooled for degradome library construction. After adaptor-trimming and genomic mapping, as done for the sRNA data, the degradome sequencing data were analysed using CleaveLand pipeline (version 3.0) (Addo-Quaye *et al.*, 2009) and PAREsnip (Folkes *et al.*, 2012). The alignment score threshold was set to 4.5 for conserved and less-conserved miRNAs (except for two *ARF* targets of miR167 and two *MYB* targets of miR858 for which the score was set to 5) and to 5 for novel and candidate miRNAs (Xia *et al.*, 2012). The apple consensus gene set from AppleGFDB and the annotation information for miRNA target genes were retrieved from the Genome Database for Rosaceae (Zhang *et al.*, 2013). Degradome data were normalized to transcripts per million values.

### Database search and scaffold locations of pear *MIKC* genes

An HMM (hidden Markov model) search was carried out in the proteome database of the Pear Genome Project (http://peargenome.njau.edu.cn:8004/default.asp?d=4&m=2) using the HMM profiles that were constructed with the MADS-box domain of the MIKC proteins from *Arabidopsis* (*Arabidopsis thaliana*). Information for other species was downloaded from the Plant Transcription Factor Database v3.0 (http://planttfdb.cbi.pku.edu.cn/index.php) (Jin *et al.*, 2014). Protein sequences encoded by the pear *MIKC* genes were searched using the HMMER 2.3.2 software package (Finn *et al.*, 2011). This procedure allowed possible mistakes in the annotations in the Pear Genome Database to be detected. The full-length pear *MIKC* gene sequences were confirmed and corrected using the 3′-RACE (Takara, Kyoto, Japan) and 5′-RACE (Clontech, Palo Alto, CA) results. The gene structures were deduced from Genoscope gene annotations, from manual annotation based on the genomic sequences in the Pear Genome Database, and from comparisons with corresponding ESTs and deduced protein sequences for homologous *MIKC* genes from *Arabidopsis* (Par̆enicová *et al.*, 2003), apple (Tian *et al.*, 2014), and grape (Díaz-Riquelme *et al.*, 2009). Scaffold locations of the pear *MIKC* genes were obtained using BLAST software 2.25 (ftp:/ncbi.nlm.nih.gro/blast/executables/release/) to align the *MIKC* sequences against the pear genome sequence.

### Characterization of MIKC sequences by 5′- and 3′-RACE and gene cloning

Finally, to validate and gain the full-length sequences of the 30 pear *MIKC* genes identified, 5′ and 3′-RACE and whole gene cloning were conducted to obtain complete, high-quality sequences of the *MIKC* genes. SMATer RACE cDNA Amplification Kit (Clontech, Palo Alto, CA) was used following the manufacturer’s instructions. A 2 μg sample of total RNA isolated from pear flower buds was used to ligate the 5′ RNA adaptors at room temperature. To amplify the full-length sequences of the *MIKC* genes, the first-strand cDNA for 5′/3′-RACE was synthesized using a SMARTer RACE cDNA Amplification Kit (Clontech) according to the manufacturer’s instructions. Pooled RNA from five different organs/tissues (leaf, bud, flower, root, and stem) served as the template. All the PCR products were ligated into the pMD18-T vector (Takara, Dalian, China) and sequenced. Specific primers were designed for nested PCR (see Supplementary Table S9 in Supplementary File 3 at *JXB* online). The 3′ and 5′ sequences were cloned and used for further analyses.

### Phylogenetic analysis

Phylogenetic and molecular evolutionary analyses were conducted using MEGA version 5 (Tamura *et al.*, 2011). To generate a phylogenetic tree, the complete sequences of the MIKC predicted proteins of pear, *Arabidopsis*, poplar, and other species shown in Table 1 and Fig. 2 were aligned using the MultAlin server (Corpet, 1988). The Neighbor–Joining method in MEGA was used to construct different trees. To estimate evolutionary distances, the proportions of amino acid differences were computed using amino acid *p*-distances. The pair-wise deletion option was used to handle gaps and missing data. The reliability of the obtained trees was tested using bootstrapping with 1 000 replicates. Phylogenetic trees were also built for MIKC proteins belonging to the TM8, AP1/FUL, and SEP subfamilies. Additional proteins from plant species other than *Arabidopsis* and poplar were included for the trees built using the TM8 and SEP protein sequences.

**Table 1.** *MIKC* genes located in pear genome

Gene name	Genome locustag	Nucleotideaccession no.	Proteinlength	Scaffoldlocation	Start	End	Strand
*PpSEP1-1*	Pbr023545.1	KP164002	246	scaffold362.0	29656	24054	+
*PpSEP1-2*	Pbr016601.1	KP164016	247	scaffold245.0	405364	399707	+
*PpSEP1-2*	Pbr016601.1	KP164016	247	scaffold362.0	29656	24054	+
*PpSEP3*	Pbr035643.1	KP164000	239	scaffold693.0	161256	166333	–
*PpSEP3*	Pbr035643.1	KP164000	239	scaffold224.0	152253	157511	–
*PpSEP4*	Pbr003650.1	KP164018	249	scaffold14.0	823203	817295	+
*PpFLC*	Pbr008076.1	KP164015	111	scaffold1479.0	9672	8911	+
*PpAP1-1*	Pbr016599.2	KP164023	265	scaffold245.0	386022	378826	+
*PpAP1-1*	Pbr016599.2	KP164023	265	scaffold362.0	10302	2812	+
*PpAP1-2*	Pbr007180.1	KP164001	255	scaffold14.0	798103	792511	+
*PpAP1-3*	Pbr029990.1	KP164004	239	scaffold51.0	538814	542536	–
*PpAG-1*	Pbr029686.2	KP164008	242	scaffold50.0	821691	815268	+
*PpAG-2*	Pbr002427.2	KP164020	243	scaffold11.0	864611	856371	+
*PpAG-3*	Pbr039503.1	KP164007	243	scaffold85.0	31027	38192	–
*PpAG-4*	Pbr000556.1	KP164009	245	scaffold1.0	3913454	3920947	–
*PpSOC1-1*	Pbr032788.1	KP164006	235	scaffold594.0	240688	248990	–
*PpSOC1-1*	Pbr032788.1	KP164006	235	scaffold1032.0	121179	128821	–
*PpSOC1-2*	Pbr032787.2	KP164011	252	scaffold1032.0	93002	97487	–
*PpSOC1-3*	Pbr039897.1	KP164003	219	scaffold867.0	76921	62046	+
*PpSOC1-3*	Pbr039897.1	KP164003	219	scaffold867.0	119095	133970	–
*PpAGL11-1*	Pbr000828.1	KP164005	223	scaffold100.0	379789	384633	–
*PpAGL11-2*	Pbr004239.1	KP164014	224	scaffold12.0	1040762	1048419	–
*PpAGL12-1*	Pbr000804.1	KP164024	202	scaffold100.0	155880	149200	+
*PpAGL12-2*	Pbr004234.1	KP164021	224	scaffold12.0	1003303	996520	+
*PpAGL17*	Pbr036758.1	KP164022	250	scaffold164.0	352520	361892	–
*PpAGL18*	Pbr002033.1	KP164010	263	scaffold107.0	267094	272146	–
*PpAGL18*	Pbr002033.1	KP164010	263	scaffold412.0	396911	402126	–
*PpBS*	Pbr022146.1	KP164017	234	scaffold895.0	59423	61625	–
*PpPI*	Pbr035294.1	KP164019	215	scaffold68.0	224867	222049	+
*PpCBM1*	Pbr029989.1	KP164012	236	scaffold51.0	527888	534330	–
*PpDAM1*	Pbr019340.1	KP164027	234	scaffold293.0	397851	387556	+
*PpDAM2*	Pbr019339.1	KP164026	227	scaffold293.0	358251	348890	+
*PpDAM3*	Pbr038022.1	KP164028	222	scaffold790.0	25713	37562	–
*PpSVP*	Pbr039693.1	KP164029	227	scaffold858.0	108158	111780	–
*PpTM8-1*	Pbr037444.1	KP164013	207	scaffold760.0	18929	21076	–
*PpTM8-1*	Pbr037444.1	KP164013	207	scaffold263.0	234794	232647	+
*PpTM8-2*	Pbr036879.1	KP164025	204	scaffold74.0	251371	248217	+

### Conserved motifs and intron/exon structure analysis

To identify shared motifs and structural divergences among the predicted full-length MADS-box proteins, the MEME online tool (http://meme.nbcr.net/meme/intro.html) was used with the following parameters: number of repetitions, any; maximum number of motifs, 6; minimum motif width, 10; and maximum motif width, 50. SMART (http://smart.embl-heidelberg.de/) and Pfam (Bateman *et al.*, 2004) were used to annotate and identify motifs. Exon–intron structural information for the *MIKC* genes was obtained from the Pear Genome Project. The DNA sequences of the *MIKC* genes were extracted from the pear genome using in-house Perl software, and the intron/exon distribution patterns were analysed using the GSDS2.0 web tool (http://gsds.cbi.pku.edu.cn).

### Real-time quantitative RT-PCR analysis

Total RNA used for the qRT-PCR analyses was extracted from lateral flower buds collected on six different dates; 15 November, 15 December, 8 January, 25 January, 15 February, and 8 March (2010/2011). Three biological replicates of 100 buds in total were used. Total RNA was extracted as described above, genomic DNA was removed with DNase I, and the total RNA concentration was measured. First-strand cDNA was synthesized from 1 μg DNA-free RNA using the Revert Aid First Strand cDNA Synthesis Kit (Fermentas, Glen Burnie, MD, USA) according to the manufacturer’s instructions. The cDNA was used as the template for qRT-PCR. The primer sequences (designed using primer 3, http://bioinfo.ut.ee/primer3-0.4.0/) are listed in Supplementary Table S10 in Supplementary File 3 at *JXB* online. The measurements were obtained using the relative quantification method and the gene transcript levels were normalized to that of the actin gene (*PpActin*, JN684184) (Liu *et al.*, 2012).

### Hierarchical clustering analysis

Genes whose transcript levels showed statistical changes related to irradiation were grouped using a two-way hierarchical clustering method in the TIGR MeV v. 3.0.1 software package (Eisen *et al.*, 1998). Pearson’s distance and average linkage clustering were used for data aggregation.

### Cloning of coding and promoter regions of *PpDAM* and *PpFT2*

The promoter regions of *PpDAM* and *PpFT2* were isolated using a Genome Walking Kit (Clontech) according to the manufacturer’s protocols. The primers for amplification of *PpFT2* were designed based on the complete cDNA sequence of *PpFT2a* [GenBank: AB571595]. The primers are listed in Supplementary Table S11 in Supplementary File 3 at *JXB* online. PCR products were analysed on 1% agarose gels. For each reaction product, a single fragment was recovered from the gels and purified using a DNA purification kit (Takara). The fragment was then ligated into the pMD18-T vector, transformed into *E. coli* DH5α competent cells (Takara), and then sequenced (Sangong, Shanghai, China).

### Yeast one-hybrid assay

The Y1H assays were performed using a Matchmaker Gold Yeast One-Hybrid System Kit (Clontech) according to the manufacturer’s protocol. The fragments of the promoters of *PpDAM* and *PpFT2* were each ligated into the pAbAi vector to generate pAbAi-bait plasmids. The whole coding regions of *PpCBF* and *PpDAM* were each ligated into the pGADT7 vector to generate the AD-*PpCBF* and AD-*PpDAM1* constructs. The primers used to clone the promoters and coding regions of *PpCBF* and *PpDAM* are listed in Supplementary Table S11 in Supplementary File 3 at *JXB* online. The pAbAi vector ligated to the *PpDAM* promoter and the *PpDAM* promoter with mutated C-repeat/DRE site were linearized and transformed into the Y1H Gold yeast strain. Transformants were selected on plates containing a selective synthetic dextrose medium lacking uracil. The AD-*PpCBF* constructs were transformed into the Y1H Gold strain harbouring pAbAi-bait and screened on SD/-Leu/AbA 150 μM plates. All transformations and screenings were performed three times. The same processes were performed for the*PpFT2* promoter and the screenings were performed on SD/-Leu/AbA 200 μM plates.

### Transient assays of gene function

Transient assays, or dual luciferase assays, were performed with tobacco (*Nicotiana benthamiana*) as reported previously (Hellens *et al.*, 2005), using pGreenII 0800-LUC and pGreenII 0029 62-SK (Hellens *et al.*, 2005). The full-length sequences of the *PpCBF* and *PpDAM1* transcription factors were individually cloned into the multiple cloning sites of pGreenII 0029 62-SK, while the promoter sequences of *PpDAM1* and *PpFT2* were combined with pGreenII 0800-LUC. The primers used for the full-length gene and promoter amplifications are described in Supplementary Table S11 in Supplementary File 3 at *JXB* online. All constructs were individually electroporated into *Agrobacterium tumefaciens* GV3101 (MP90). Infiltrations, transient expression analysis, and determination of LUC and REN enzyme activities were conducted. Three days after infiltration, LUC (Firefly luciferase) and REN (Ranilla luciferase) activities were analysed using a Dual-Luciferase Reporter Assay System (Promega, Madison, WI, USA). Measurements were carried out using a Modulus Luminometer (Promega) in three independent experiments with at least four biological replicates for each assay. In a separate experiment, *PpCBF* was infiltrated into the tobacco abaxial leaf surface in pairs containing the *PpDAM* promoter fragment, and *PpDAM* was infiltrated into the tobacco abaxial leaf surface in pairs containing the *PpFT2* promoter fragment (A-type).

## Results

### Dormancy status of lateral flower buds in pear

**Fig. 1.**
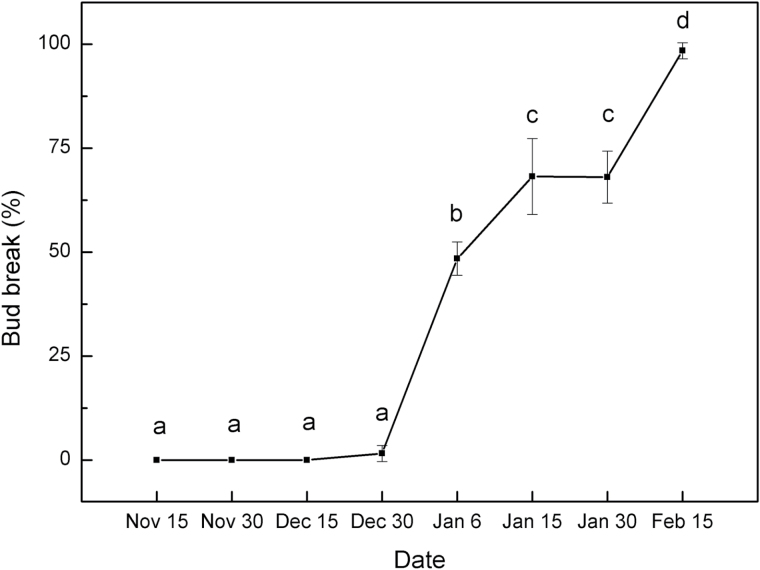
Bud break percentage of ‘Suli’ pear after 21 d of forcing conditions. Dormant shoots of field-grown ‘Suli’ pear trees were collected from 15 November 2010 to 15 February 2011, and kept in water in a phytotron at day/night temperatures of 25±1/18±1 °C, with a 12h photoperiod of white light (320 μmol photon m^–2^ s^–1^), and 75% humidity. Percentage bud break was assessed after 21 d using 12 shoots per sampling period. Error bars show the standard deviation of three biological replicates. Means with the same letter among stages are not significantly different (*P* ≤ 0.05).

To measure the transcript profiles of miRNA and *MIKC* genes during dormancy transition in pear, the dormancy status of the lateral flower buds was first defined. The dormancy status of buds was measured on excised one-year-old shoots of ‘Suli’ pear (*Pyrus pyrifolia* white pear group) on eight collection dates. Almost no bud breaks were observed on shoots sampled from 15 November to 30 December, but more than 50% of the buds had broken on shoots collected after 15 January (Fig. 1). Thus, the lateral flower buds sampled from 15 November to 30 December were determined as being in the endo-dormancy phase and those sampled from 15 January to 15 February in the eco-dormancy phase.

### Identification, annotation, and location of pear *MIKC* genes in genome scaffolds

A total of 30 *MIKC* genes were identified in the pear genome and mapped to defined positions on the scaffolds (Table 1). Full-length cDNA sequences of the 30 *MIKC* genes were determined using 5′- and 3′-RACE (rapid amplification of cDNA ends). The pear *MIKC* genes were named based on their assignment to previously established *MIKC* subfamilies and numbered when several genes were identified for a same subfamily (Table 1). Based on the available sequence information, three of the *MIKC* sequences were identified as *DAM* genes, one as an *SVP*, and one as a *FLOWERING LOCUS C* (*FLC*) gene (Table 1). Of the 30 *MIKC* genes, 29 (96.7%) contained more than seven introns; *PpSEP3.2* contained the most introns (nine), and *SUPPRESSOR OF CONSTANS1-3* (*SOC1-3*) had the longest intron (see Supplementary Fig. S1 in Supplementary File 1 at *JXB* online). Two gene copies were found in each of the *SEPALLATA1-2* (*SEP1-2*), *SEP3*, *APETALA1-1* (*AP1-1*), *SOC1-1*, *SOC1-3*, *AGAMOUS-LIKE18* (*AGL18*), and *TM8-1* subfamilies, which were located in different regions of the pear genome (Table 1). The 30 *MIKC* genes were distributed on 27 scaffolds in the Pear Genome Database (Table 1); three genes were located on scaffold362.0, and two genes were located on each of scaffold100.0, scaffold1032.0, scaffold12.0, scaffold 245.0, scaffold293.0, scaffold51.0, and scaffold867.0 (Table 1). The 30 pear *MIKC* genes were subjected to further analyses.

### Phylogenetic analysis of *MIKC* genes

**Fig. 2.**
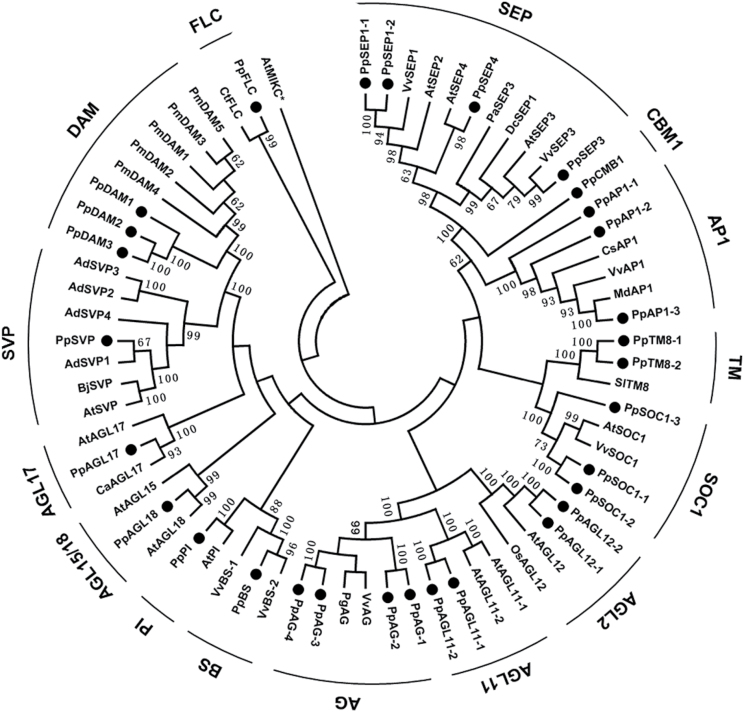
Phylogenetic tree of the MIKC gene family in pear. The phylogenetic tree was constructed based on a multiple sequence alignment of predicted full-length MIKC protein sequences of *Pyrus pyrifolia* (Pp), *Actinidia deliciosa* (Ad), *Arabidopsis* (At), *Brassica juncea* (Bj), *Coffea arabica* (Ca), *Citrus sinensis* (Cs), *Citrus trifoliata* (Ct), *Dendrobium crumenatum* (Dc), *Malus domestica* (Md), *Oryza sativa* (Os), *Platanus acerifolia* (Pa), *Panax ginseng* (Pg), *Prunus mume* (Pm), *Solanum lycopersicum* (Sl), and *Vitis vinifera* (Vv). Numbers at nodes are percentage bootstrap values based on Neighbor–Joining analysis. The groups were marked with bold bars outside of the tree. The MIKC proteins identified in the pear genome were marked with black dots.

To examine the phylogenetic relationships among the pear *MIKC* genes and group them within the established subfamilies, a Neighbor–Joining phylogenetic tree was constructed based on a multiple sequence alignment of the predicted full-length MIKC protein sequences of pear, *Arabidopsis*, poplar, and peach (Fig. 2). The 30 pear *MIKC* genes clustered into 15 subfamilies (Fig. 2). The *DAM*, *SOC1*, and *AP1* subfamilies each contained three pear homologues, both the *AG* and *SEP* subfamilies contained four pear homologues, and each of the *AGL11*, *AGL12*, *AP1*, and *TM* subfamilies contained two pear homologues (Fig. 2). Among the remaining five genes, *PpPI* was grouped in the *PISTILLATA* (*PI*) subfamily, while *PpSVP, PpAGL17, PpAGL18*, and *PpFLC* were unambiguously grouped with orthologous genes from other species. Therefore, the pear genome seemed to have only one *SVP*, *AGL17*, *AGL18*, *FLC*, and *PI* gene. Each pear gene in the *AGL17* and *AGL18* subfamilies had one orthologous gene in *Arabidopsis*, suggesting that no duplication events occurred among these genes after pear and *Arabidopsis* diverged, and that these genes might play similar roles in pear and *Arabidopsis*. However, there were four homologues of *AGAMOUS* (*AG*) in pear (*PpAG-1*, *PpAG-2*, *PpAG-3*, and *PpAG-4*) but only two *AGs* in *Arabidopsis*, implying that the pear *AG* subfamily may have undergone a recent duplication event (Fig. 2).

### Identification of conserved protein motifs and *cis*-acting elements in promoters

To assess the diversity and similarity of motif composition among the pear *MIKC* genes, the MEME tool was used (Bailey *et al.*, 2009) to identify motifs in the 30 predicted MIKC protein sequences. Six motifs were identified (see Supplementary Fig. S2 in Supplementary File 1 at *JXB* online); motif 1 specified the MADS domain while a combination of motifs 2, 4, and 5 specified the K domain. All of the MIKC proteins contained motif 1 and motif 2-type MADS domains. Although the K domain was specified by a combination of three motifs (2, 4, and 5), many of the pear *MIKC* genes contained only two of these motifs, either motifs 2 and 4 or motifs 2 and 5, indicating that the K domain was moderately conserved (see Supplementary Fig. S2 in Supplementary File 1 at *JXB* online). It was found that the same or closely related subfamilies shared similar motifs and motif distributions, which supported the classification of the pear *MIKC* genes.

To analyse the promoter sequences of the *MIKC* genes, the 1kb upstream sequences and the 5′ UTRs were extracted for all 30 genes to create the promoter constructs listed in Supplementary File 2 at *JXB* online. Candidate *cis*-acting elements in these promoter sequences were predicted using the website tools at PlantCARE (http://bioinformatics.psb.ugent.be/webtools/plantcare/html/). Intriguingly, it was found that the promoters of both *PpDAM1* and *PpDAM3* had a CBF transcription factor (AB826494) binding site, namely the C-repeat/dehydration responsive element (C-repeat/DRE) (see Supplementary Figs S2, S4, and S5 in Supplementary File 1 at *JXB* online).

### Expression analysis of pear *MIKC* genes

**Fig. 3.**
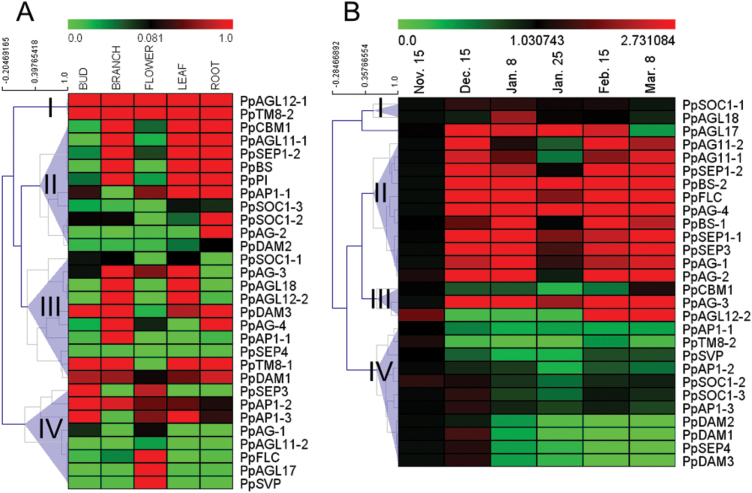
Transcript profiles of pear *MIKC* genes. Transcript analyses were performed by qRT-PCR. (A) Transcript profiles of pear *MIKC* genes in different pear organs/tissues. (B) Transcript profiles of pear *MIKC* genes during bud dormancy transition.

*MIKC* genes are thought to be involved in regulating dormancy, flowering time, and the specification of reproductive organ identity. As shown in Fig. 3A, there was a wide range in the transcript levels of the 30 pear *MIKC* genes among the five representative vegetative and reproductive organs/tissues of pear. Transcripts of *PpAGL12-1* and *PpTM8-2* were detected in all five organs/tissues, and these two genes showed the highest transcript levels among the 30 *MIKC* genes. The *DAM* subfamily genes and *PpTM8-1* showed high transcript levels in the bud, stem, and root, but very low transcript levels in the flower. *PpAP1-2*, *PpAP1-3*, and *PpSEP3* showed relatively high transcript levels in the bud. Transcripts of *PpAGL17*, *PpAGL12-1*, *PpTM8-2*, *PpSVP*, and *PpFLC* were mainly detected in the flower. There were low transcript levels of *AG* subfamily genes. In summary, most of the *MIKC* subfamilies were transcribed predominantly in specific tissue(s), and genes belonging to the same subfamily did not always show the same transcriptional patterns among the five organs/tissues (Fig. 3A).

To identify the *MIKC* genes that may be involved in regulating dormancy transition, the transcript profiles of *MIKC* genes were analysed in different stages of bud dormancy by real-time quantitative RT-PCR (qRT-PCR). Using MeV software (Eisen *et al.*, 1998) and gene-wise expression normalization, the 30 *MIKC* genes were classified into four gene expression groups according to the chronological stages of bud dormancy: endo-dormancy, eco-dormancy, and late-expressed genes (Fig. 3B). In the endo-dormancy stage, transcripts of 18 genes in the *DAM*, *AGL*, *SEP*, *B-sister* (*BS*), *AG*, *SOC*, and *FLC* subfamilies were detected. Among them, *PpDAM1*, *PpDAM3*, *PpSOC1-3*, *PpSEP4*, and *PpAP1-3* transcripts seemed to accumulate at similar levels; their transcript levels peaked at the endo-dormancy stage on 15 December, and then decreased on 8 January and 8 March. Among all the *MIKC* genes, *PpSVP*, *PpAP1-1*, *PpAP1-2*, *PpCBM1*, and *PpTM8-2* showed relatively low transcript levels at all stages of bud dormancy. The transcriptional patterns of *PpSOC1-1* and *PpAGL18* differed from those of all the other *MIKC* genes and peaked at the eco-dormancy stage on 8 January, and then deceased rapidly. The late-expression group contained only one gene, *PpAGL12-2*, whose transcripts were not detected until 15 February (Fig. 3B).

### Interaction between *PpDAM* promoters and *Pp*CBF

**Fig. 4.**
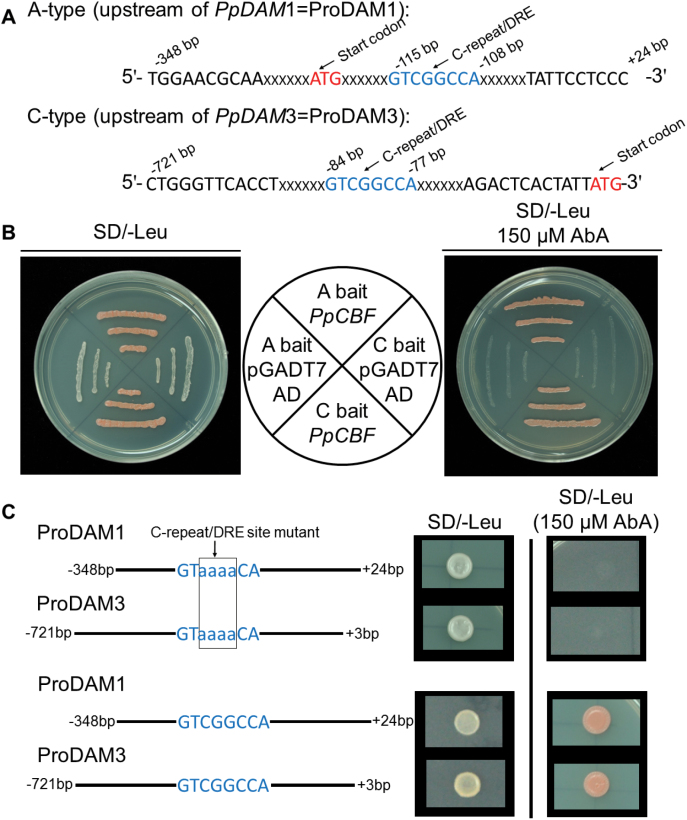
Interaction between *Pp*CBF and *PpDAM* promoter as determined by Y1H assay. (A) Upstream regions of *PpDAM* A- and C-type promoters showing location of C-repeat/DRE transcription factor binding site. (B) Y1H assays showing interaction between *Pp*CBF and *PpDAM* promoters. (C) The promoter of *PpDAM* with mutated C-repeat/DRE site was synthesized artificially and was inserted into pAbAi plasmid for Y1H assays. The pAbAi vector ligated to the promoter of *PpDAM* with non-mutated C-repeat/DRE site as a positive control. Y1H assays showed interaction between *Pp*CBF and promoters of *PpDAM* with mutated C-repeat/DRE site and non-mutated C-repeat/DRE site. (This figure is available in colour at *JXB* online.)

**Fig. 5.**
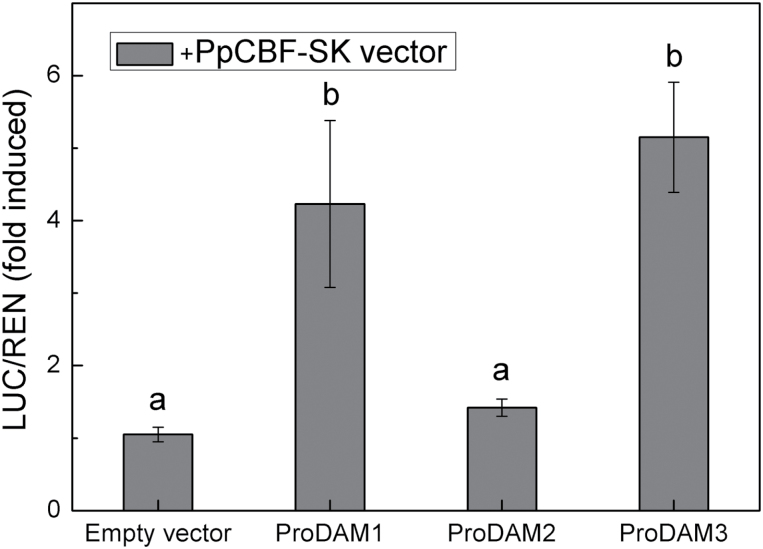
Dual luciferase transient expression assays to probe functions of promoters and transcription factors. Interaction between *PpDAM* promoters and *Pp*CBF in tobacco leaves. The activity of firefly and renilla luciferase in tobacco leaves was detected 3 d after infiltration. Error bars show standard error (SE) of three independent experiments with at least four replicate reactions. Means with the same letter among different injections are not significantly different (*P* ≤ 0.05).

**Fig. 6.**
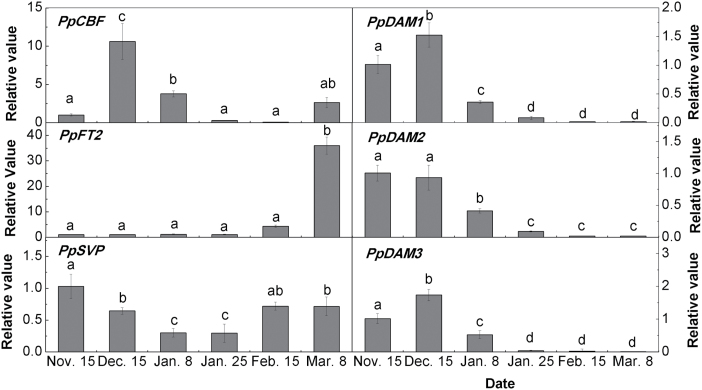
Expression levels of dormancy-associated genes in pear flower buds during different bud dormancy stages. Error bars show the standard deviation of three biological replicates. Means with the same letter among stages are not significantly different (*P* ≤ 0.05).

A yeast one-hybrid (Y1H) assay was conducted to detect the interaction between *Pp*CBF (DDBJ accession number AB826494) and the *PpDAM* promoter (ProDAM). The promoter *cis*-elements analysis predicted that a CBF-binding site (C-repeat/DRE element) was present in ProDAM1 and ProDAM3, but not in ProDAM2 (see Supplementary Figs S2, S4, and S5 in Supplementary File 1 at *JXB* online). One fragment was cloned from each of ProDAM1, ProDAM2, and ProDAM3 and designated as A-type (–348 to +24bp), B-type (–680 to –12bp) and C-type (–680 to –12bp), respectively (Fig. 4A; see Supplementary Fig. S6 in Supplementary File 1 at *JXB* online). The Y1H assay showed that *Pp*CBF could associate with ProDAM1 and ProDAM3, but not with ProDAM2 and promoter of *PpDAM* with the mutated C-repeat/DRE site (Fig. 4B, C; see Supplementary Fig. S6 in Supplementary File 1 at *JXB* online), suggesting that *Pp*CBF might interact with the C-repeat/DRE sites in ProDAM1 and ProDAM3 (Fig. 4). The interaction of *Pp*CBF and ProDAM was further identified in tobacco. Dual luciferase assays indicated that when *PpCBF* was co-transformed with ProDAM, the activities of the ProDAM1 and ProDAM3 promoters were increased by 4.2 times and 5.1 times, respectively, compared with the negative control transformed with only the empty vector (Fig. 5). During bud dormancy transition, *PpCBF*, *PpDAM1*, and *PpDAM3* showed similar transcription patterns. Their transcript levels increased and peaked from 15 November to 15 December during endo-dormancy, and then decreased rapidly (Fig. 6). However, *PpDAM2* expression was down-regulated during the transition of bud dormancy from 15 November to 15 February. These data indicated that the cold response transcription factor *Pp*CBF promoted the expression of *PpDAM1* and *PpDAM3* by binding with the C-repeat/DRE site in ProDAM.

### Interaction between the *PpFT2* promoter and *Pp*DAM

**Fig. 7.**
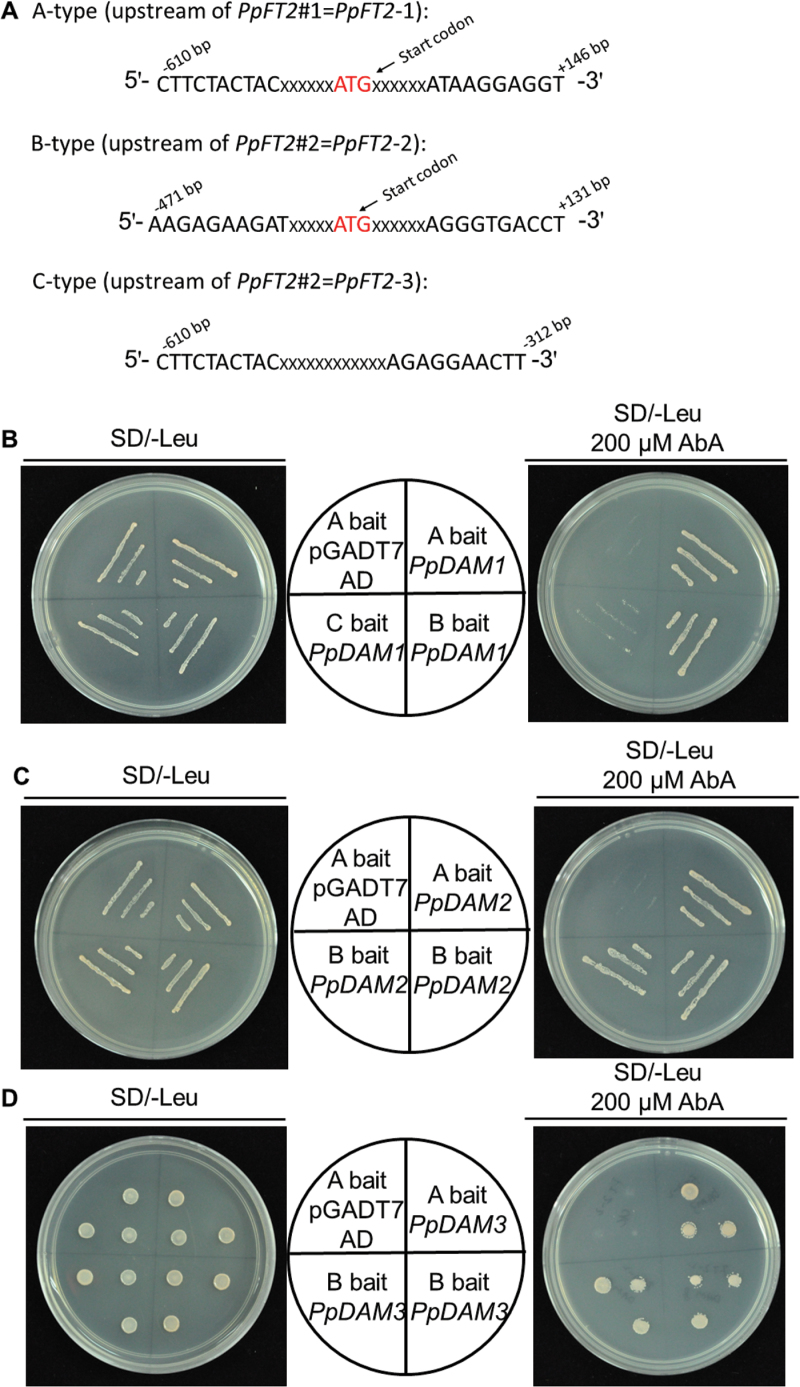
Interaction between *Pp*DAM and promoter of *PpFT2* as determined by Y1H assay. (A) Upstream regions of *PpFT2* A-, B- and C-type promoters. (B) Y1H assays showing interaction between *Pp*DAM and *PpFT2* promoters. Note that PpDAMs associated with the –312 to +131bp fragment of ProFT2. (This figure is available in colour at *JXB* online.)

**Fig. 8.**
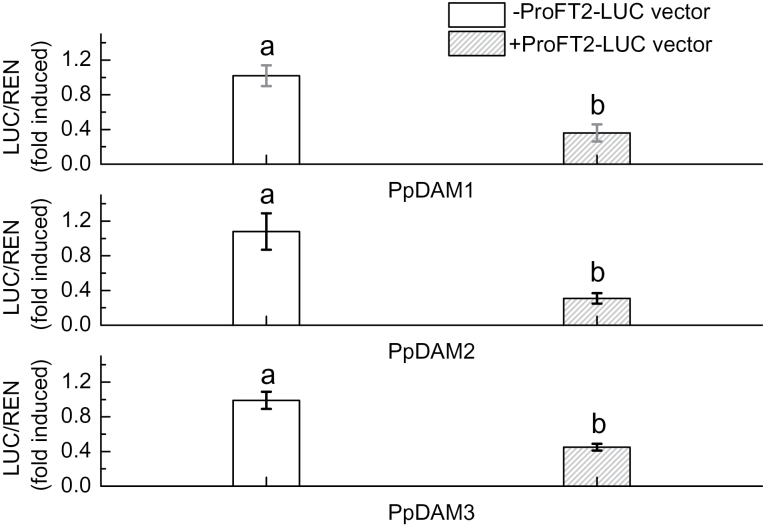
Dual luciferase transient expression assays to probe functions of promoters and transcription factors. The activity of firefly and renilla luciferase in tobacco leaves was detected 3 d after infiltration. Error bars show standard error (SE) of three independent experiments with at least four replicate reactions. (A) Interaction between *PpFT2* promoter and *Pp*DAM1 in tobacco leaves. (B) Interaction between *PpFT2* promoter and *Pp*DAM2 in tobacco leaves. (C) Interaction between *PpFT2* promoter and *Pp*DAM3 in tobacco leaves. Means with the same letter among different injections are not significantly different (*P* ≤ 0.05).

A Y1H assay was also carried out to detect the interaction between *Pp*DAM and the *PpFT2* (AB571595) promoter (ProFT2). ProFT2 was divided into three fragments: A-type (–610 to +146bp), B-type (–471 to +131bp), and C-type (–610 to –312bp) (Fig. 7A). The Y1H assay showed that all three *Pp*DAMs were able to associate with either A-type or B-type, but not C-type (Fig. 7). The results showed that *Pp*DAM associated with the –312 to +131bp fragment of ProFT2 (Fig. 7A). The interaction between *Pp*DAM and ProFT2 *in vitro* was studied in yeast, while their interaction *in vivo* was identified in tobacco. Dual luciferase assays indicated that when each *PpDAM* was co-transformed with ProFT2 (A-type), the activity of ProFT2 was more than 2.2 times lower than that in the negative control transformed with only the empty vector (Fig. 8). During the bud dormancy state transition, the transcript level of *PpDAM* decreased rapidly from 15 December to 15 February (Fig. 6). By contrast, *PpFT2* transcripts were first detected on 15 February, and the transcript level significantly increased from 15 February to 8 March (Fig. 6). These data indicated that *Pp*DAM inhibited the expression of *PpFT2* by binding with the upstream region of *PpFT2*.

### Identification and expression profiles of conserved and less-conserved miRNAs in pear during bud dormancy

**Fig. 9.**
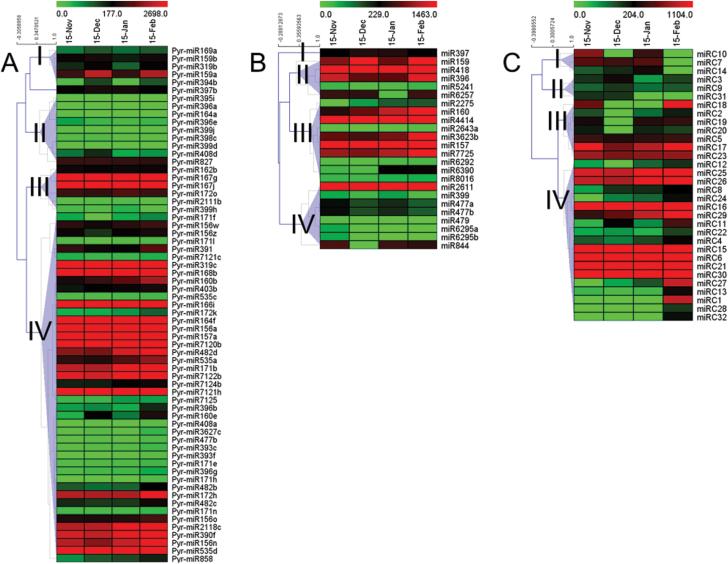
Expression profiles of conserved and less-conserved miRNAs in pear flowering buds during bud dormancy. (A) Expression profiles of conserved miRNAs. (B) Expression profiles of less-conserved miRNAs. (C) Expression profiles of pear-specific miRNAs. Detailed list of miRNAs used in this figure can be found in Supplementary Tables S2 and S4 in Supplementary File 3 at *JXB* online.

To identify miRNAs responsive to bud dormancy in pear at the various stages of dormancy (para-, endo-, and eco-dormancy), four small RNA (sRNA) libraries were constructed from total RNA extracted from pear flowering buds during bud dormancy. A total of 63.4 million reliable reads were obtained from four sRNA libraries. Most of these reads (approximately 71% of redundant reads and 86% of unique reads) had at least one perfect match to the pear genome (see Supplementary Table S1 in Supplementary File 3 at *JXB* online). The sRNAs from each library shared similar length distribution patterns (see Supplementary Table S1 in Supplementary File 3 at *JXB* online), with 24-nucleotide sRNAs being the most abundant (>50%) followed by 21-nucleotide sRNAs (see Supplementary Table S2 in Supplementary File 3 at *JXB* online). The miRNAs were identified by mapping the unique sRNA sequences that mapped perfectly to the pear genome to miRBase 21.0 (Kozomara and Griffiths-Jones, 2014) with a maximum of two bases-mismatch. As a result, 39 conserved miRNA families were identified (Fig. 9A; see Supplementary Table S2 in Supplementary File 3 at *JXB* online). The identified miRNAs bore a canonical stem-loop structure in their pre-miRNA (precursor) sequences (see Supplementary Table S3 in Supplementary File 3 at *JXB* online). The expression levels of the conserved miRNAs, as reflected by the normalized reads (reads per million genome-matched reads; RPM), showed large variations among the different bud dormancy stages (Fig. 9A; see Supplementary Table S2 in Supplementary File 3 at *JXB* online).

To understand the expression patterns of conserved miRNAs that were significantly differentially expressed at different stages in pear dormancy (see Supplementary Table S2 in Supplementary File 3 at *JXB* online), a cluster analysis of the miRNAs expression patterns was performed based on three comparisons (15 November versus 15 December, 15 December versus 15 January, and 15 January versus 15 February) (Fig. 9A). The cluster analysis identified four major clusters of expression patterns (Fig. 9A). Approximately 68% of the conserved miRNAs fell into group IV; their expressions were up-regulated during bud endo-dormancy and release. This group contained seven known development-related miRNA families with differential expression: miR156, miR157, miR160, miR171, miR172, miR482, and miR535 (Fig. 9A; see Supplementary Table S2 in Supplementary File 3 at *JXB* online). Members of these seven miRNA families are involved mainly in plant development and stress responses, as well as in the plant hormone signalling pathway. The expressions of miR160b, miR482d, and miR535a increased dramatically during bud dormancy, with high expression levels from 15 January to 15 February. This pattern suggested that the expressions of these three miRNAs might be affected by chilling; therefore, they may play roles as regulators during endo-dormancy maintenance and release (Fig. 9A; see Supplementary Table S2 in Supplementary File 3 at *JXB* online). Some of the remaining miRNAs fell into groups II and III, which showed no obvious or only slight changes in expression levels during bud dormancy (Fig. 9A; see Supplementary Table S2 in Supplementary File 3 at *JXB* online). In some cases, different members of the same miRNA family showed different expression patterns. For instance, miR172k and miR172h were clustered in group IV, while miR172o was in group III based on its expression pattern (Fig. 9A). This result implied that different members of the same family may have distinct functions in bud dormancy.

A total of 24 miRNAs or miRNA families that had a standard stem-loop structure were also identified in pear. There were designated as less-conserved miRNAs (Fig. 9B; see Supplementary Table S2 in Supplementary File 3 at *JXB* online). These miRNAs were not identified widely in either the angiosperm or Coniferophyta lineages. When compared with the conserved miRNAs, most of the less-conserved miRNAs showed lower expression levels. The most notable exception was miR4414, which was expressed at an abundance of >8 000 RPM at every stage (Fig. 9B; see Supplementary Table S2 in Supplementary File 3 at *JXB* online). These 24 less-conserved miRNAs were significantly differentially expressed (Fig. 9B; see Supplementary Table S2 in Supplementary File 3 at *JXB* online) during dormancy transition, and were divided into four groups (Fig. 9B). The largest group (group II) comprised 10 (41.6%) genes that showed up-regulated expression during bud dormancy transition. Their expression patterns were similar to that of the conserved miRNAs in group IV (Fig. 9A), suggesting that these miRNAs might have similar roles in regulating bud dormancy. Group I miRNAs (miR159, miR418, miR396, miR5241, and miR6257) were down-regulated from 15 November to 15 January, and then up-regulated. Group IV miRNAs (miR2611, miR399, miR477, miR479, miR6295, and miR844) were down-regulated from 15 November to 15 December, and then up-regulated (Fig. 9B; see Supplementary Table S1 in Supplementary File 3 at *JXB* online).

**Fig. 10.**
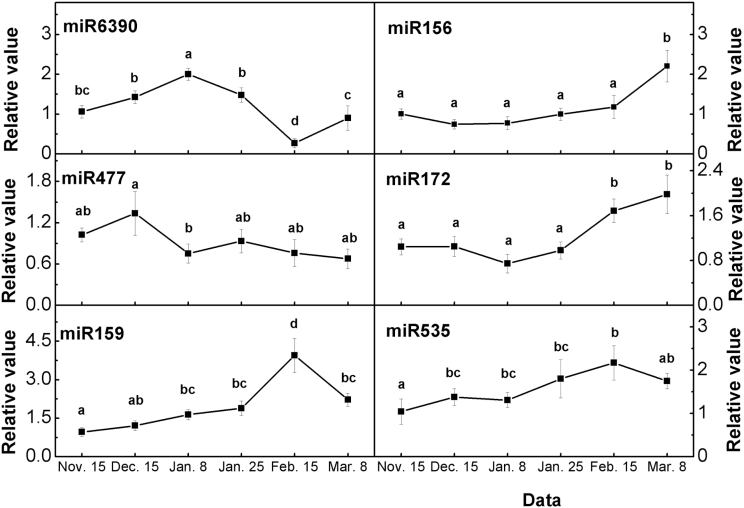
qRT-PCR validations of the expression levels of miRNAs in pear flower buds during bud dormancy. Error bars show the standard deviation of three biological replicates. Means with the same letter among stages are not significantly different (*P* ≤ 0.05).

To validate the miRNA RPM data, qRT-PCR analyses were performed to detect selected miRNAs in pear buds at six stages during bud dormancy (Fig. 10). The expression pattern of eight miRNAs detected by qRT-PCR were similar to the relative abundances of the sequenced miRNAs detected in these four tissues, thereby validating our gene transcript analysis (Fig. 10; see Supplementary Table S2 in Supplementary File 3 at *JXB* online).

### Pear-specific miRNAs

After excluding the sRNA reads homologous to known miRNAs (two or fewer mismatches, miRBase 21.0) and other non-coding sRNAs (Rfam 10 (Griffiths-Jones *et al.*, 2005)), the pre-miRNAs of the remaining 18- to 24-nucleotide-long sRNAs were subjected to rigorous secondary structural analysis using RNAfold software (http://nhjy.hzau.edu.cn/kech/swxxx/jakj/dianzi/Bioinf4/miRNA/miRNA1.htm). Pre-miRNAs with a canonical stem-loop structure were analysed further through a series of stringent filtering strategies to ensure that they met established criteria commonly used to identify candidate miRNAs. As a result, 32 miRNA candidates derived from 46 loci (see Supplementary Table S4 in Supplementary File 3 at *JXB* online) were considered to be novel pear miRNAs; 25 were 21-nucleotides long and four were 23-nucleotides long (see Supplementary Table S4 in Supplementary File 3 at *JXB* online). Precursors forming hairpin structures are listed in Supplementary Table S5 in Supplementary File 3 at *JXB* online. A cluster analysis of the expression patterns of the candidate miRNAs (Fig. 9C) revealed four major clusters. The largest group (group IV) contained 19 (59.4%) genes showing up-regulated expression from 15 November to 15 February. Among them, miRC8, miRC12, miRC24, miRC27, and miR29 were markedly up-regulated, indicating that they may play important roles in regulating bud dormancy. The second largest group (group II) contained seven (21.9%) genes, and their expressions were down-regulated from 15 November to 15 December and then up-regulated.

### Identification and annotation of targets of pear miRNAs

**Fig. 11.**
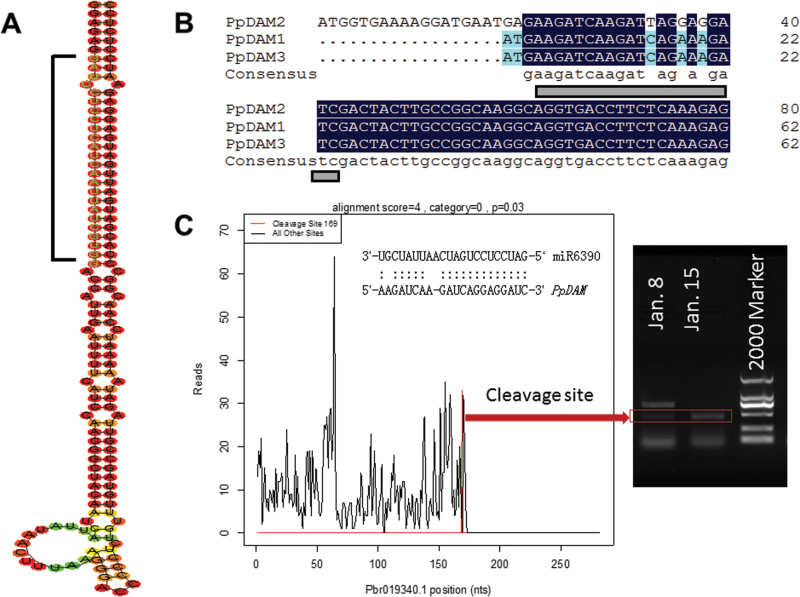
The secondary structures of miR6390 precursor and its target genes *PpDAM*s. (A) Predicted secondary structure of pre-miR6390. (B) The pairing between miR6390 and its target sites within *PpDAM*s is illustrated. (C) Target plot (t-plot) for miR6390 targets confirmed by degradome sequencing and the cleavage site was confirmed by the 5′-RACE nested PCR. (This figure is available in colour at *JXB* online.)

To identify the gene targets for the conserved, less-conserved, and pear-specific miRNAs, degradome sequencing was performed to generate a total of 18 million short reads representing the 5′ ends of uncapped, poly-adenylated RNAs. Approximately 69% of the unique reads aligned perfectly (no mismatches) to the pear transcriptome. Eighty-one targets in five categories were identified (0–4) (see Supplementary Table S6 in Supplementary File 3 at *JXB* online). Among the 62 targets for conserved miRNA families, 13 were in category 0, which represented the most abundant degradome tags corresponding to the cleavage site and matching cognate transcripts, and 29 were in category 2, which had the second most abundant degradome tags. The number of targets of different miRNAs ranged from 1 to 14 (see Supplementary Table S6 in Supplementary File 3 at *JXB* online) and miRNAs that targeted members of a gene family usually had more targets. For example, miR172 could target three members of the AP2-like factor gene family, miR166 could target three BZIP-domain transcription factors, and miR156 and miR157 could target members of the *SPL* family (see Supplementary Table S6 in Supplementary File 3 at *JXB* online) to regulate plant growth and the abiotic stress response. The auxin signalling-related miRNAs miR393 and miR408 might play a role in the dormancy process by adjusting auxin levels. In addition, miR408, whose target gene *ZEP* participates in ABA synthesis, was identified in pear bud during dormancy transition (see Supplementary Table S7 in Supplementary File 3 at *JXB* online). The miR858 family was predicted to repress the conserved *MYB* genes that have been implicated in anthocyanin synthesis. In particular, miR6390 could target *PpDAM* (Fig. 11; see Supplementary Table S6 in Supplementary File 3 at *JXB* online), which might be involved in regulating bud dormancy. The cleavage site of miR6390-targeted *PpDAM1* was confirmed by the 5′ RACE nested PCR (Fig. 11D). Therefore, miR6390-regulated *PpDAM* might define an endogenous dormancy pathway in pear.

Based on the degradome sequencing data, miR6390 was predicted to bind to a site in the *PpDAM* mRNA (Fig. 11B), and the target plot of miR6390 confirmed the binding site (Fig. 11C). The secondary hairpin structure of the pre-miR6390 sequence is shown in Fig. 11A. The qRT-PCR analysis showed that miR6390 and its target *PpDAM1* had opposite transcription patterns during bud dormancy (Figs 6, 8). This result inferred that miR6390 might play a role in bud dormancy by targeting and degrading *PpDAM* (Fig. 11).

## Discussion

### Genome-wide identification and transcriptional analysis of *MIKC* genes during bud dormancy transition

Thirty *MIKC* genes were identified in the pear genome and they were divided into 15 subfamilies in the phylogenetic analysis (Fig. 2). Fewer *MIKC* genes were identified in pear than in *Arabidopsis* (39), cucumber (40), grapevine (38), and poplar (55). Par̆enicová *et al*. (2003) proposed that *MIKC* genes might play similar regulatory roles in several plant development processes. Typically, the pear *MIKC* genes (see Supplementary Fig. S2 in Supplementary File 1 at *JXB* online) comprised exons 1–6 and conserved C-terminal motifs, and were similar to the structure of MADS-box genes found in other plants (Johansen *et al.*, 2002), indicating that the MADS-box family, and particularly *MIKC* genes, is highly conserved in plants (Par̆enicová *et al.*, 2003). Genes in seven subfamilies (*SEP1-2*, *SEP3*, *AP1-1*, *SOC1-1*, *SOC1-3*, *AGL18*, and *TM8-1*) (23.3% of the 30 *MIKC* genes) had two duplicate copies that shared the same promoter, but the genes were located on different scaffolds in the Pear Genome Database (Table 1). Similar gene duplication events have been found in the apple genome, where there were two or more copies of 58.2% of the MADS-box genes (Tian *et al.*, 2014). Genome duplication events are thought to have occurred throughout the process of plant genome evolution (Crooks *et al.*, 2004). The location of the pear *MIKC* genes in different scaffold regions (Table 1), similar to the diverse locations of *MIKC* genes in *Arabidopsis* (Par̆enicová *et al.*, 2003), rice (Arora *et al.*, 2007), grapevine (Díaz-Riquelme *et al.*, 2009), and apple (Tian *et al.*, 2014), suggested that *MIKC* genes were widely distributed in the genome of the common ancestor of monocots and eudicots. It has been reported that a relatively recent (30–45 million years ago) genome-wide duplication event resulted in the transition of nine ancestral chromosomes to 17 chromosomes in pear (Wu *et al.*, 2013), which might explain why there were duplicate copies of many pear *MIKC* genes.

*MIKC* genes that regulate dormancy transition in peach, apricot, and leafy spurge belong mainly to the *DAM*, *SVP*, *SOC,* and *FLC* subfamilies (Li *et al.*, 2009; Horvath *et al.*, 2010; Wu *et al.*, 2012; Zhou *et al.*, 2013). In perennial species, *DAM* genes, which are closely related to *SVP*, have been identified as major regulators of the endo-dormancy transition (Horvath *et al.*, 2002; Li *et al.*, 2009). In pear, the *DAM* subfamily had three members, which have been reported to be involved in regulating bud dormancy (Liu *et al.*, 2012). In this study, three *DAM* genes showed the same expression patterns during bud dormancy in the lateral flower bud, consistent with the results of a previous study (Liu *et al.*, 2012). None of these three genes was expressed in the pear flower (Fig. 3A), suggesting that DAM may not affect flowering. In addition, there were lower transcript levels of *PpDAM2* than *PpDAM1* and *PpDAM3* in the leaf, bud and shoot (Fig. 3A), suggesting that *PpDAM2* might play a minor role in regulating bud dormancy. In *Arabidopsis*, *FLC* is a key floral regulator in the MIKC subfamily that acts by suppressing *FT* expression (Samach *et al.*, 2000). Extended cold temperatures cause a modification of the chromatin structure around the *FLC* promoter and epigenetically inhibit the transcription of *FLC* (Sung and Amasino, 2004). Unlike the case in *Arabidopsis*, the expression of the *FLC*-like gene (*PpFLC*) was up-regulated towards endo-dormancy release in pear, indicating that cold accumulation did not repress *FLC* expression (Fig. 3B). A similar result was also described for trifoliate orange (Zhang *et al.*, 2009), suggesting that *PpFLC* might not act as a key regulator in regulating dormancy transition by chromatin remodelling as reported for *Arabidopsis* (Sung and Amasino, 2004). Besides, it was found that the transcriptional patterns of *PpSOC1-2*, *PpSOC1-3*, *PpAP1-3*, and *PpSEP4* were similar to those of *DAM* genes (Fig. 3B), indicating that these genes might also play important roles in controlling dormancy transition. Expression analyses of *MIKC* genes have suggested that floral morphological differentiation accompanies dormancy transition. For instance, homologues of *AP1*, which determines sepal development in *Arabidopsis* (Gustafson-Brown *et al.*, 1994), have been identified from various species including lily (*Lilium longiflorum*) (Chen *et al.*, 2008), soybean (*Glycine max*) (Chi *et al.*, 2011), and longan (*Dimocarpus longan*) (Winterhagen *et al.*, 2013). Overexpression of the *AP1* gene in transgenic soybean plants was shown to cause early flowering (Chi *et al.*, 2011). Our results suggested that MIKC proteins involved in floral organ determination might be closely associated with endo-dormancy release. However, more studies are needed to confirm this speculation. The results presented here provide the framework for further studies on the roles of *MIKC* genes in dormancy transition. Also, these findings may motivate evolutionary biologists to study the evolution of this important transcription factor family in plants and other organisms.

### Genome-wide identification and characterization of miRNAs and their expression during bud dormancy transition

MiRNAs are critical post-transcriptional regulators of gene expression during the plant response to cold stress (Chinnusamy *et al.*, 2007). However, the regulation of miRNAs in flower buds in response to cold winters is poorly understood. Recently, high-throughput sequencing has provided powerful data for understanding miRNA-mediated regulatory networks in plants. In apple, 165 miRNAs belonging to 56 families have been recorded in AppleGFDB, the Apple Gene Function and Gene Family DataBase v1.0 (http://www.applegene.org/mirna.asp). In peach, 117 conserved miRNAs and 186 novel miRNA candidates have been identified (Luo *et al.*, 2013). In the present study, 185 conserved miRNAs, 24 less-conserved miRNAs, and 32 novel miRNAs were identified in pear flower buds, more than the 186 conserved microRNAs identified in the pear genome by bioinformatics methods and reported in our previous study (Niu *et al.*, 2013). However, little is known about the roles of miRNAs in regulating pear bud dormancy. A comprehensive analysis of miRNAs during endo-dormancy maintenance and release at a genome-wide level has been presented here. These analyses revealed the transcriptional patterns of the miRNAs involved in this process (Fig. 9). In addition, a set of miRNAs with specific expression patterns has been identified. Two age-regulated miRNAs, miR156 and miR172, which were previously found to be involved in regulating the timing of sensitivity in the response to vernalization in *Arabidopsis* (Chen, 2004), were also shown to control the meristem cell fate transition in maize (Chuck *et al.*, 2008) and the dormancy phase change in poplar (Ding *et al.*, 2014). In our dataset, miR156 and miR172 showed similar expression patterns during bud dormancy (Figs 9A, 11), implying that these miRNAs may be regulatory factors that can be recruited to control dormancy transition. The expression patterns of miR160b, miR482d, miR535a, and miR171b were similar to those of miR156 and miR172; therefore, they may play similar roles in regulating dormancy.

Overall, 81 targets of 19 miRNA families were detected in pear by degradome sequencing, giving an average of 4.26 targets per miRNA (see Supplementary Table S6 in Supplementary File 3 at *JXB* online), similar to values reported in other studies (Ding *et al.*, 2014). In the present study, some important transcription factors were found to be targeted by miRNAs. For example, *AP2*, an important transcription factor that controls flowering and seed development in *Arabidopsis*, was predicted to be the target gene of miR172 in pear bud in this study and our previous study (Chen, 2004). In addition, some hormone pathway genes were identified as the targets of pear miRNAs. For instance, miR393 targeted the auxin receptor 1 mRNA (Pbr022779.1) and miR408 targeted the *AUXIN RESPONSE FACTOR* (*ARF*, Pbr021104.1) and *zeaxanthin epoxidase* (*ZEP*, Pbr005027.1) genes (see Supplementary Table S6 in Supplementary File 3 at *JXB* online). In rice, *ZEP* (*OsABA1*) was identified as the key regulator in ABA synthesis, and mutants that had lost *OsABA1* function displayed low ABA levels, and almost no increase in ABA levels under drought conditions (Agrawal *et al.*, 2001). It is suspected that miR408 may play an important role in regulating bud dormancy by controlling the level of ABA in pear buds. Besides the universal targets such as *ARF*, *ZEP*, *AP2*, and *SPL* genes, which are involved in regulating bud dormancy transition in trees (Li *et al.*, 2009; Sasaki *et al.*, 2011), *DAM* genes were identified as possible targets of miR6390 for the first time in this study. Our results have provided a comprehensive analysis of miRNAs in buds during dormancy and new evidence of the miRNAs that may be involved in regulating this biological process.

### Genetic network and molecular model for regulation of endo-dormancy transition

Previous studies have shown that CBFs play a key role in regulating dormancy and the low-temperature response (Kendall *et al.*, 2011). *CBF*s are believed to be regulated by the transcription factor INDUCER of CBF EXPRESSION 1 (ICE1), which is present at normal growing temperature but is either activated by, or interacts with, cold-activated proteins (Thomashow, 2001). In this study, *PpCBF* was up-regulated more than 10-fold during endo-dormancy (Fig. 6). Also, a CBF-binding site (C-repeat/DRE) was present in the promoters of *PpDAM1* and *PpDAM3* (see Supplementary Figs S3, S4, and S5 in Supplementary File 1 at *JXB* online). Although a CBF-binding site was also found in the leafy spurge *DAM1* promoter (Horvath *et al.*, 2010), an interaction between CBF and *DAM1* was not reported in that study. The *in vitro* Y1H assay and *in vivo* transient expression analysis showed that *Pp*CBF activated the transcription of *PpDAM1* and *PpDAM3* by binding to their promoters (Figs 4, 5). The results are consistent with recent reports (Saito *et al.*, 2015). Therefore, the cold response factor *Pp*CBF may play a key role in maintaining endo-dormancy by directly up-regulating transcription of *PpDAM1* and *PpDAM3* (Fig. 6). Also, *Arabidopsis* mutants lacking *CBF*s had low levels of DELAY OF GERMINATION1 (DOG1) and GA2 OXIDASE (GA2ox6) in dry seeds (Kendall *et al.*, 2011). DOG1 and GA2ox6 have been reported to be involved in regulating gibberellin (GA) and ABA levels, and were found to be central factors in the temperature response of seed dormancy (Kendall *et al.*, 2011). The ABA response locus *ABI* encodes an AP2 domain protein, and *ABI4* showed the highest sequence homology to genes encoding the class of proteins including the tobacco ABA response element binding protein (ABRE) and the *Arabidopsis* CBF1 protein (Finkelstein *et al.*, 1998). There were three ABRE binding sites in the promoter of *PpDAM3* (see Supplementary Fig. S5 in Supplementary File 1 at *JXB* online). Therefore, ABI, which has an AP2 domain, might also affect *DAM* expression, suggesting that ABA could also be involved in regulating endo-dormancy maintenance via an interaction with *DAM*s.

**Fig. 12.**
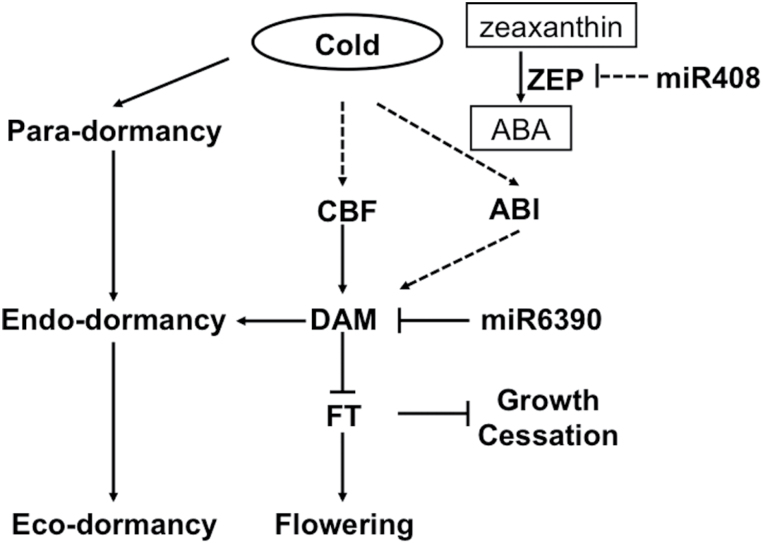
Proposed model of genetic factors that may affect dormancy transition in pear.Solid arrows/bars indicate genes, hormones, metabolites, or environmental conditions that have been proven to induce/inhibit targets; dashed arrows/bars indicate those that have been proposed but not yet confirmed in induction/inhibition of targets in this study. Short-term chilling in autumn activates the accumulation of CBF, which directly promotes *DAM* expression; DAM subsequently inhibits *FT2* expression to induce endo-dormancy and miR6390 degrades *DAM* genes to release endo-dormancy. Short-term cold also induced ABA accumulation that might enhance the endo-dormancy by activating the *ABI* gene.

*FT* is mainly expressed in source leaves in response to environmental conditions that promote flowering; however, there is some evidence that it is also expressed in young leaves, shoot apices, and dormant buds (Horvath *et al.*, 2008; Wilkie *et al.*, 2008). Chromatin immunoprecipitation assays were performed using DAM-like protein-specific antibodies to demonstrate that DAM or related proteins likely bind to cryptic and/or conserved CArG boxes in the promoter regions of *FT* genes (Hao *et al.*, 2015). There is also some evidence that members of the *FT* gene family are involved in altering endo-dormancy. The direct or indirect over-expression of *FT*s in poplar has resulted in the failure of the buds to enter endo-dormancy (Böhlenius *et al.*, 2006). It has been hypothesized that, in dormant tissue, induction of *DAM* expression may down-regulate *FT* during the initiation of growth cessation and/or endo-dormancy (Horvath *et al.*, 2008, 2010). Overexpression of leafy spurge *DAM1* in transgenic *Arabidopsis* resulted in down-regulated *FT* expression and delayed flowering comparing to the wild type (Horvath *et al.*, 2010). Our *in vitro* Y1H assay and *in vivo* transient expression analyses showed that *Pp*DAM1 inhibited the expression of *PpFT2* by binding to its promoter. Transcripts of *PpFT2* were not detected during the bud dormancy process, but *PpFT2* transcription was significantly up-regulated after dormancy release (15 February) (Figs 6, 7, 8). These results are consistent with the hypothesis that the expression of *DAM* inhibited the expression of *PpFT2* by binding to its promoter during bud dormancy, and that both DAM and FT2 played crucial roles in regulating bud dormancy maintenance and release in pear (Fig. 12).

Post-transcriptional regulatory mechanisms such as pre-miRNA splicing, mRNA export, and miRNA-directed mRNA degradation, also play important roles in cold stress responses (Sunkar and Zhu, 2004). In poplar, miR156 and miR172 showed opposite expression patterns in the cambial dormancy–active growth transition (Ding *et al.*, 2014). In addition, miR160, which was reported to be involved in the auxin signalling pathway, was expressed specifically during endo-dormancy release by chilling, consistent with our gene transcription results (Ding *et al.*, 2014). Besides the known miRNAs, our results have revealed novel miRNAs and their possible target genes that may contribute to regulating the dormant–active growth transition. These findings may provide new insights into the regulatory mechanisms of dormancy transition in trees. Furthermore, based on the degradome sequence data, it was found that miR6390 targeted *PpDAM* genes and that miR6390 and *PpDAM* showed opposite expression patterns, indicating that miR6390 might play a crucial role in dormancy release via degradation of *PpDAM* (Fig. 11). However, more experiments are needed to verify the role of miRNAs in regulating dormancy.

By combining the above findings, a model is proposed of a *PpDAM* gene-centred molecular mechanism that could regulate bud dormancy maintenance and release in pear (Fig. 12). In this model, short-term exposure to cold induces *PpCBF* expression in pear buds, and the *Pp*CBF protein then activates *PpDAM1* and *PpDAM3* expression for the bud to enter endo-dormancy. Meanwhile, *Pp*DAM inhibits *PpFT2* expression to maintain endo-dormancy. The up-regulated expression of miR6390 gradually degrades DAM products, further inducing expression of *PpFT2*. Then, bud dormancy release occurs and the bud is ready to break under suitable temperatures.

### Accession numbers

The sequencing data obtained in this work have been submitted to the NCBI under the accession numbers listed in Table 1.

### Statistical analysis

Least significant differences (α=0.05) were calculated for mean separations using the Data Processing System (version 7.05; Zhejiang University, Hangzhou, China).

## Supplementary data

Supplementary data can be found at *JXB* online.

Supplementary File 1: Supplementary Figs S1 to S6

Supplementary Fig. S1. Intron/exon structure and distribution patterns in pear genome of 30 pear *MIKC* genes.

Supplementary Fig. S2. Distribution of conserved motifs in pear MIKC proteins identified using MEME search tool.

Supplementary Fig. S3. Predicted *cis*-acting elements in *PpDAM1* promoter region.

Supplementary Fig. S4. Predicted *cis*-acting elements in *PpDAM2* promoter region.

Supplementary Fig. S5. Predicted *cis*-acting elements in *PpDAM3* promoter region.

Supplementary Fig. S6. Interaction between *Pp*CBF and promoter of *PpDAM2* as determined by Y1H assay; note that PpCBF cannot bind to the promoter of *PpDAM2* in Y1H.

Supplementary File 2: Predicted promoter sequences of *MIKC* genes of ‘Suli’ pear

Supplementary File 3: Supplementary Tables S1 to S11

Supplementary Table S1. Read statistics in four libraries.

Supplementary Table S2. Detailed list of homologous sequences for known miRNAs.

Supplementary Table S3. Known miRNAs with good stem-loop structure.

Supplementary Table S4. Detailed list of pear-specific miRNAs found in ‘Suli’ pear.

Supplementary Table S5. Novel and candidate miRNAs with good stem-loop structure.

Supplementary Table S6. Targets of pear miRNAs (or miRNA families; detailed list).

Supplementary Table S7. The metabolism pathways that were potentially regulated by miRNAs using the KEGG pathway analysis.

Supplementary Table S8. Primer sequences used to amplify miRNAs.

Supplementary Table S9. Primers used to clone the *MIKC* genes.

Supplementary Table S10. Primer sequences used to amplify genes (or families; detailed list).

Supplementary Table S11. Primers for amplification of full-length promoters and TFs.

Supplementary Data

## Abbreviations:

ARF: auxin response factor
CBF: C-repeat binding factors
DAM: dormancy-associated MADS-box gene
FT: FLOWERING LOCUS T
*MIKC* genes: *MIKC^C^-type MADS-box* genes
miRNA: microRNA
PCR: polymerase chain reaction
qRT-PCR: quantitative real-time PCR
RPM: reads per million genome-matched reads
sRNA: small RNA
Y1H: yeast one-hybrid.
