# Supplementary material for: Dormancy-associated MADS-box genes and microRNAs jointly control dormancy transition in pear (Pyrus pyrifolia white pear group) flower bud
Source: J Exp Bot. 2015 Oct 14;67(1):239–57. doi: 10.1093/jxb/erv454 (PMC4682432; doi:10.1093/jxb/erv454)
Supplement: Supplementary Data [file supp_67_1_239__index.html]

Dormancy-associated MADS-box genes and microRNAs jointly control dormancy transition in pear (Pyrus pyrifolia white pear group) flower bud — Dormancy-associated MADS-box genes and microRNAs jointly control dormancy transition in pear (Pyrus pyrifolia white pear group) flower bud — Supplementary Data 

# Dormancy-associated MADS-box genes and microRNAs jointly control dormancy transition in pear (*Pyrus pyrifolia* white pear group) flower bud

## Supplementary Data

Data files

- Supplementary\_Figures\_S1\_to\_S6.pdf - Supplementary Data
- Predicted\_promoter\_sequences\_of\_MIKC\_genes\_of\_Suli\_pear.fasta - Supplementary Data
- Supplementary\_Tables\_S1\_to\_S11.xlsx - Supplementary Data
