# Supplementary material for: Dormancy-associated MADS-box genes and microRNAs jointly control dormancy transition in pear (Pyrus pyrifolia white pear group) flower bud
Source: J Exp Bot. 2015 Oct 14;67(1):239–57. doi: 10.1093/jxb/erv454 (PMC4682432; doi:10.1093/jxb/erv454)
Supplement: Supplementary Data [file supp_erv454_Supplementary_Figures_S1_to_S6.pdf]

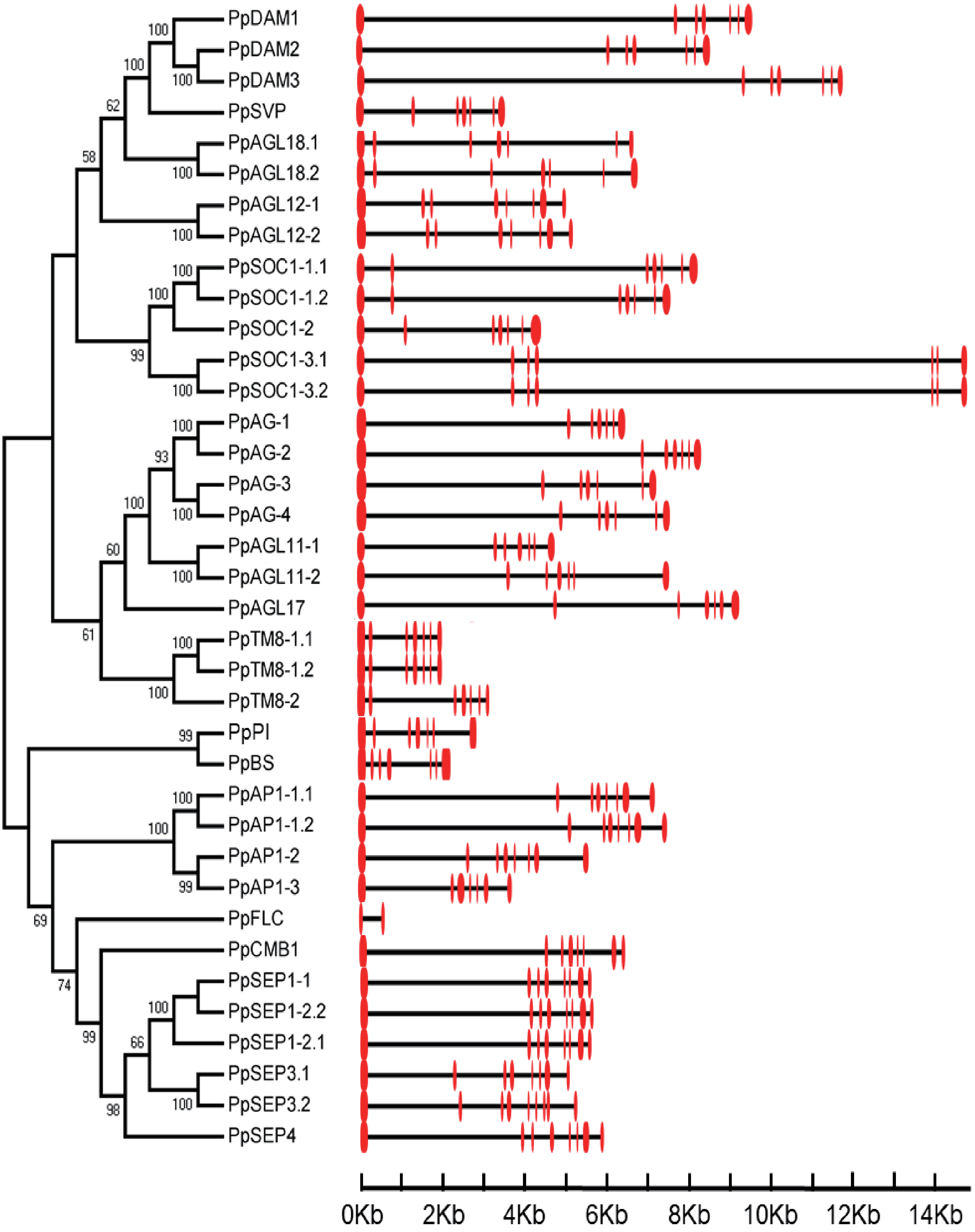
PpDAM1
PpDAM2
PpDAM3
PpSVP
PpAGL18.1
PpAGL18.2
PpAGL12-1
PpAGL12-2
PpSOC1-1.1
PpSOC1-1.2
PpSOC1-2
PpSOC1-3.1
PpSOC1-3.2
PpAG-1
PpAG-2
PpAG-3
PpAG-4
PpAGL11-1
PpAGL11-2
PpAGL17
PpTM8-1.1
PpTM8-1.2
PpTM8-2
PpPI
PpBS
PpAP1-1.1
PpAP1-1.2
PpAP1-2
PpAP1-3
PpFLC
PpCMB1
PpSEP1-1
PpSEP1-2.2
PpSEP1-2.1
PpSEP3.1
PpSEP3.2
PpSEP4
0Kb
2Kb
4Kb
6Kb
8Kb
10Kb
12Kb
14Kb

Figure S1

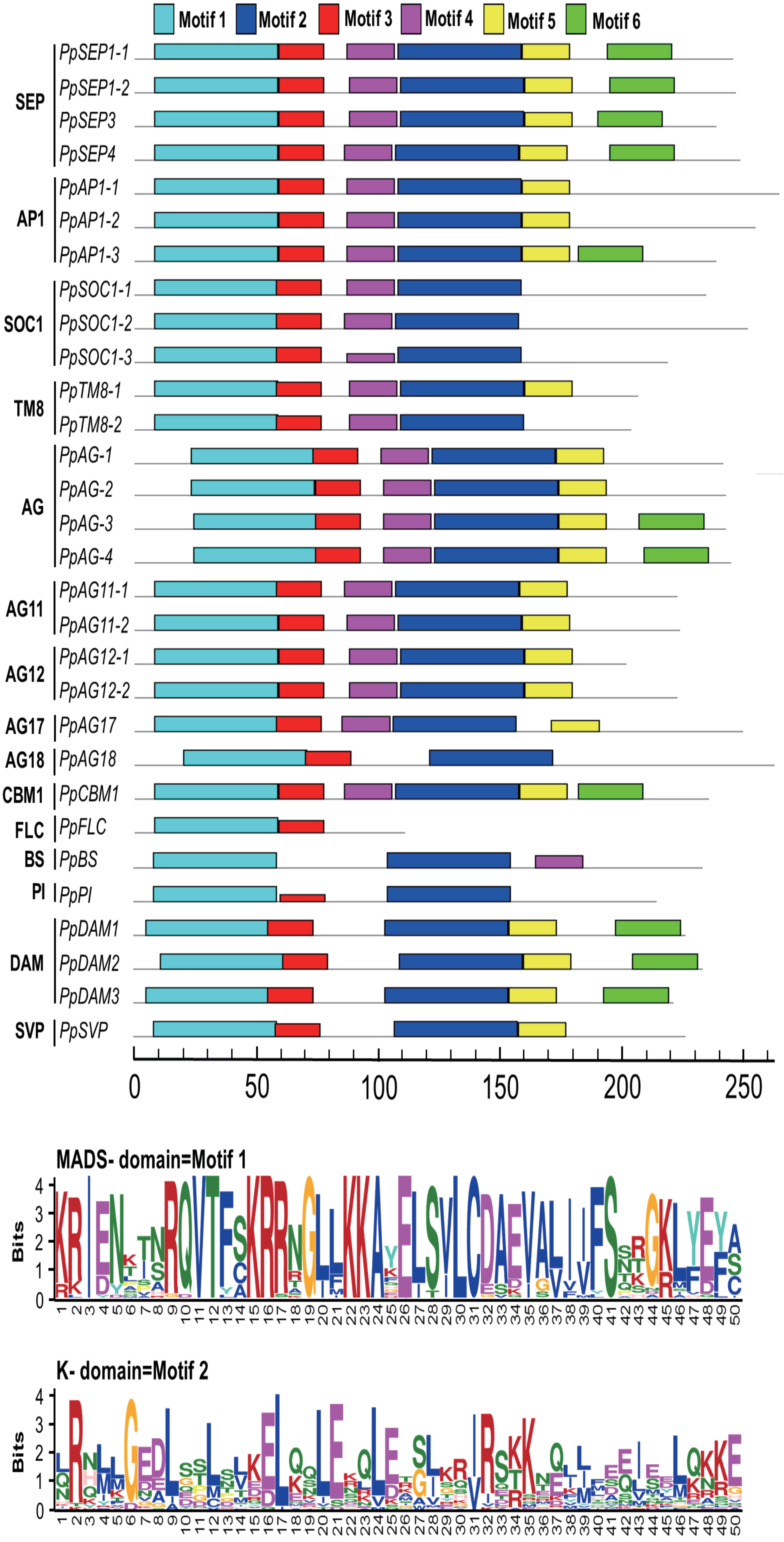
Motif 1
Motif 2
Motif 3
Motif 4
Motif 5
Motif 6
SEP
PpSEP1-1
PpSEP1-2
PpSEP3
PpSEP4
AP1
PpAP1-1
PpAP1-2
PpAP1-3
SOC1
PpSOC1-1
PpSOC1-2
PpSOC1-3
TM8
PpTM8-1
PpTM8-2
AG
PpAG-1
PpAG-2
PpAG-3
PpAG-4
AG11
PpAG11-1
PpAG11-2
AG12
PpAG12-1
PpAG12-2
AG17
PpAG17
AG18
PpAG18
CBM1
PpCBM1
FLC
PpFLC
BS
PpBS
PI
PpPI
DAM
PpDAM1
PpDAM2
PpDAM3
SVP
PpSVP
0
50
100
150
200
250
MADS- domain=Motif 1
Bits
K- domain=Motif 2
Bits

Figure S2

```
+ CTTAAAACAA ACCAACGCCT AAACAAAATC AACATTCAGT CGCATGAAGA GATTTCAGAG AAGAGGAAGG
- GAATTTTGTT TGGTTGCGGA TTTGTTTTAG TTGTAAGTCA GCGTACTTCT CTAAAGTCTC TTCTCCTTCC

+ GAGGAATAGA AGTACCGACC ATAATTTATT TATTTATTAT AAATAAATAA ATAAATAAAA GCATATGAAG
- CTCCTTATCT TCATGGCTGG TATTAAATAA ATAAATAATA TTTATTTATT TATTTATTTT CGTATACTTC

+ AGATTGCACT GCAGGGAAGA CGAGAGGGCC GGCCGGGATG GCAACGGTGG AAGAGGGTTT GGCCGACGGT
- TCTAACGTGA CGTCCCTTCT GCTCTCCCGG CCGGCCCTAC CGTTGCCACC TTCTCCCAAA CCGGCTGCCA

+ GGATGGCCGG TGTTGCAGCG ATTGCTGCTA CCGCTGCTCT TTAGGATAAA GAAGTGAGAG AGACGAGGTG
- CCTACCGGCC ACAACGTCGC TAACGACGAT GGCGACGAGA AATCCTATTT CTTCACTCTC TCTGCTCCAC

+ GATGTGACGA CACCTAAAAA GAAATAAACC GAGAATCCCC ACTCAGTTAG ATATGAACAA ATCTAAGAGA
- CTACACTGCT GTGGATTTTT CTTTATTTGG CTCTTAGGGG TGAGTCAATC TATACTTGTT TAGATTCTCT

+ TTCCATATTT TTACTGTCGT CCATTCCACA TATATTATGA ACGGTGGTGA TCGCATTCAT CACTCATCAC
- AAGGTATAAA AATGACAGCA GGTAAGGTGT ATATAATACT TGCCACCACT AGCGTAAGTA GTGAGTAGTG

+ GCCAACTGAT TTGCGTTCCA CGTTTCTTAC CTTAAAAATA TCCAAAAATA GGAAAGAGAA ACAAACAGTT
- CGGTTGACTA AACGCAAGGT GCAAAGAATG GAATTTTTAT AGGTTTTTAT CCTTTCTCTT TGTTTGTCAA

+ GATATTAAAG CGAGATATAT CTTATTTTTC TATAAATAGG AAATTTAATT CGCCAACCTA TCCTTTCTAG
- CTATAATTTC GCTCTATATA GAATAAAAAG ATATTTATCC TTTAAATTAA GCGGTTGGAT AGGAAAGATC

+ AACAACCGGC AGCACCCGAA CTCTCTCTCT CCTCTCTGTT CTTCTCTTTC CAAAATATCT TTGGTTTCCT
- TTGTTGGCCG TCGTGGGCTT GAGAGAGAGA GGAGAGACAA GAAGAGAAAG GTTTTATAGA AACCAAAGGA

+ TTCACTGCTC TGCAAATTTC CCAAAACTTC AGACCTGAAA CTCCCCGACG AAGGTAAACA AATAACAACG
- AAGTGACGAG ACGTTTAAAG GGTTTTGAAG TCTGGACTTT GAGGGGCTGC TTCCATTTGT TTATTGTTGC

+ GAAAACCCAG TAAATAATTT ATGTATGGTT TTTTTTTTTC TTCTTCTCTT CGATTGGTGG TAAATACAAA
- CTTTTGGGTC ATTTATTAAA TACATACCAA AAAAAAAAAG AAGAAGAGAA GCTAACCACC ATTTATGTTT

+ ATACTTTAGG CTTGTTGGGC AGTTGTTTGT GGTTGGTTGG TTTTTCTGGG TTTTGATTTT CTTATTTAGA
- TATGAAATCC GAACAACCCG TCAACAAACA CCAACCAACC AAAAAGACCC AAAACTAAAA GAATAAATCT

+ TCTGAGATGC TAGGGTTTGA GGTCAATATC TTTAAACTTA TAAAAATACC CAGAAAATAT ACGGTAGTTT
- AGACTCTACG ATCCCAAACT CCAGTTATAG AAATTTGAAT ATTTTTATGG GTCTTTTATA TGCCATCAAA

+ TTGAGTTTGT GAACAGTTAT ATATGTGAAA AGTGCATGGT TGGTTTATTT TTATTTATTT GAATTAATTG
- AACTCAAACA CTTGTCAATA TATACACTTT TCACGTACCA ACCAAATAAA AATAAATAAA CTTAATTAAC

+ TTTTTGAAGG GGTAGGAAGA
- AAAAACTTCC CCATCCTTCT
```

+ 5UTR Py-rich stretch

+ AAGAA-motif

+ ACE

Site Name	Organism	Position	Strand	Matrix score.	sequence	function
ACE	Petroselinum hortense	437	-	7	ACGTGGA	cis-acting element involved in light responsiveness
ACE	Petroselinum crispum	770	-	9	CTAACGTATT	cis-acting element involved in light responsiveness

+ AE-box

Site Name	Organism	Position	Strand	Matrix score.	sequence	function
AE-box	Arabidopsis thaliana	477	+	8	AGAAACAA	part of a module for light response

+ ARE

Site Name	Organism	Position	Strand	Matrix score.	sequence	function
ARE	Zea mays	9	-	6	TGGTTT	cis-acting regulatory element essential for the anaerobic induction
ARE	Zea mays	951	+	6	TGGTTT	cis-acting regulatory element essential for the anaerobic induction
ARE	Zea mays	726	+	6	TGGTTT	cis-acting regulatory element essential for the anaerobic induction
ARE	Zea mays	622	+	6	TGGTTT	cis-acting regulatory element essential for the anaerobic induction
ARE	Zea mays	808	+	6	TGGTTT	cis-acting regulatory element essential for the anaerobic induction

+ Box 4

Site Name	Organism	Position	Strand	Matrix score.	sequence	function
Box 4	Petroselinum crispum	973	+	6	ATTAAT	part of a conserved DNA module involved in light responsiveness

+ Box-W1

Site Name	Organism	Position	Strand	Matrix score.	sequence	function
Box-W1	Petroselinum crispum	861	-	6	TTGACC	fungal elicitor responsive element

+ C-repeat/DRE

Site Name	Organism	Position	Strand	Matrix score.	sequence	function
C-repeat/DRE	Arabidopsis thaliana	200	+	8	TGGCCGAC	regulatory element involved in cold- and dehydration-responsiveness

+ CAAT-box

Site Name	Organism	Position	Strand	Matrix score.	sequence	function
CAAT-box	Hordeum vulgare	143	-	4	CAAT	common cis-acting element in promoter and enhancer regions
CAAT-box	Arabidopsis thaliana	753	-	5	CCAAT	common cis-acting element in promoter and enhancer regions
CAAT-box	Brassica rapa	643	+	5	CAAAT	common cis-acting element in promoter and enhancer regions
CAAT-box	Hordeum vulgare	977	-	4	CAAT	common cis-acting element in promoter and enhancer regions
CAAT-box	Brassica rapa	338	+	5	CAAAT	common cis-acting element in promoter and enhancer regions
CAAT-box	Brassica rapa	967	-	5	CAAAT	common cis-acting element in promoter and enhancer regions
CAAT-box	Brassica rapa	689	+	5	CAAAT	common cis-acting element in promoter and enhancer regions
CAAT-box	Hordeum vulgare	864	+	4	CAAT	common cis-acting element in promoter and enhancer regions
CAAT-box	Hordeum vulgare	231	-	4	CAAT	common cis-acting element in promoter and enhancer regions
CAAT-box	Glycine max	976	-	5	CAATT	common cis-acting element in promoter and enhancer regions
CAAT-box	Brassica rapa	429	-	5	CAAAT	common cis-acting element in promoter and enhancer regions

+ CATT-motif

Site Name	Organism	Position	Strand	Matrix score.	sequence	function
CATT-motif	Zea mays	403	+	6	GCATTC	part of a light responsive element

+ CCAAT-box

Site Name	Organism	Position	Strand	Matrix score.	sequence	function
CCAAT-box	Hordeum vulgare	182	+	6	CAACGG	MYBHv1 binding site
CCAAT-box	Hordeum vulgare	696	+	6	CAACGG	MYBHv1 binding site

+ CGTCA-motif

Site Name	Organism	Position	Strand	Matrix score.	sequence	function
CGTCA-motif	Hordeum vulgare	285	-	5	CGTCA	cis-acting regulatory element involved in the MeJA-responsiveness

+ G-Box

Site Name	Organism	Position	Strand	Matrix score.	sequence	function
G-Box	Pisum sativum	439	+	6	CACGTT	cis-acting regulatory element involved in light responsiveness

+ G-box

Site Name	Organism	Position	Strand	Matrix score.	sequence	function
G-box	Zea mays	439	+	6	CACGTT	cis-acting regulatory element involved in light responsiveness

+ GAG-motif

Site Name	Organism	Position	Strand	Matrix score.	sequence	function
GAG-motif	Arabidopsis thaliana	580	-	7	AGAGAGT	part of a light responsive element

+ L-box

Site Name	Organism	Position	Strand	Matrix score.	sequence	function
L-box	Lycopersicon esculentum	949	-	11	AAATTAACCAAC	part of a light responsive element

+ MBS

Site Name	Organism	Position	Strand	Matrix score.	sequence	function
MBS	Arabidopsis thaliana	324	-	6	TAACTG	MYB binding site involved in drought-inducibility
MBS	Arabidopsis thaliana	924	-	6	TAACTG	MYB binding site involved in drought-inducibility
MBS	Arabidopsis thaliana	486	-	6	CAACTG	MYB binding site involved in drought-inducibility
MBS	Arabidopsis thaliana	423	+	6	CAACTG	MYB binding site involved in drought-inducibility
MBS	Arabidopsis thaliana	790	-	6	CAACTG	MYB binding site involved in drought-inducibility

+ O2-site

Site Name	Organism	Position	Strand	Matrix score.	sequence	function
O2-site	Zea mays	417	-	9	GTTGACGTGA	cis-acting regulatory element involved in zein metabolism regulation

+ Sp1

Site Name	Organism	Position	Strand	Matrix score.	sequence	function
Sp1	Zea mays	69	-	5	CC(G/A)CCC	light responsive element

+ TATA-box

+ TCA-element

Site Name	Organism	Position	Strand	Matrix score.	sequence	function
TCA-element	Nicotiana tabacum	353	+	9	CCATCTTTTT	cis-acting element involved in salicylic acid responsiveness
TCA-element	Brassica oleracea	739	-	9	GAGAAGAATA	cis-acting element involved in salicylic acid responsiveness
TCA-element	Brassica oleracea	597	-	9	GAGAAGAATA	cis-acting element involved in salicylic acid responsiveness

+ TCCACCT-motif

+ TCT-motif

Site Name	Organism	Position	Strand	Matrix score.	sequence	function
TCT-motif	Arabidopsis thaliana	445	+	6	TCTTAC	part of a light responsive element

+ TGACG-motif

Site Name	Organism	Position	Strand	Matrix score.	sequence	function
TGACG-motif	Hordeum vulgare	285	+	5	TGACG	cis-acting regulatory element involved in the MeJA-responsiveness

+ Unnamed__1

+ Unnamed__11

+ Unnamed__2

+ Unnamed__3

+ Unnamed__4

+ W box

+ WUN-motif

Site Name	Organism	Position	Strand	Matrix score.	sequence	function
WUN-motif	Brassica oleracea	528	-	9	AAATTTCCT	wound-responsive element

+ circadian

Site Name	Organism	Position	Strand	Matrix score.	sequence	function
circadian	Lycopersicon esculentum	614	-	9	CAAAGATATC	cis-acting regulatory element involved in circadian control

Figure S3

```
+ ATTTATTTAA ATTAATAAAA TCATATGAAG ATATTGCTTT GCAGAGAAGA CGAAGGGGCC GGGATGGCAA
- TAAATAAATT TAATTATTTT AGTATACTTC TATAACGAAA CGTCTCTTCT GCTTCCCCGG CCCTACCGTT

+ TGGTGGAAGA GGGTTTGGAG AATGCGGCTA CTGAAACTCG GGGGTTGGGG TTTGGGGAGT TTTGTTTCAC
- ACCACCTTCT CCCAAACCTC TTACGCCGAT GACTTTGAGC CCCCAACCCC AAACCCCTCA AAACAAAGTG

+ AAGTGGGAGT GAGTATTGCC AGCAAGTGTT GCAGCGATCG CTGCTACCGC TGCTGTTTGG GATAAAGAAG
- TTCACCCTCA CTCATAACGG TCGTTCACAA CGTCGCTAGC GACGATGGCG ACGACAAACC CTATTTCTTC

+ TGAGAGAGAC GAAGTGGGTG AGGAAAATCA ACGGTGTGGT ACGTAAAAAG AATTAAACGT AGAATCCCCA
- ACTCTCTCTG CTTCACCCAC TCCTTTTAGT TGCCACACCA TGCATTTTTC TTAATTTGCA TCTTAGGGGT

+ GCCTTACTGT CGTCCAGTCC ACATATTATA GACGGTCGTG ATCGCATTCA TGACTCATCA CACCAACTGA
- CGGAATGACA GCAGGTCAGG TGTATAATAT CTGCCAGCAC TAGCGTAAGT ACTGAGTAGT GTGGTTGACT

+ TTTGTGTTCC GCGTTTCTAA CCTTATGTTC CCAAAAAATA TCTAAAAAGA CAAAAAGGCA CAAACAGTGA
- AAACACAAGG CGCAAAGATT GGAATACAAG GGTTTTTTAT AGATTTTTCT GTTTTTCCGT GTTTGTCACT

+ TAACCAAAGG AAGATATGTC CTATTTTTCC ATAATTAGGA AAATTAATTT AGCAACCTTT CCTTTCTTGA
- ATTGGTTTCC TTCTATACAG GATAAAAAGG TATTAATCCT TTTAATTAAA TCGTTGGAAA GGAAAGAACT

+ ACTGCCGGCA GCATCCCAAC TCTTTCTCAT CTCTCTTCTT CTCTTTCCAG AATTGCTGGC TTTTCTTTCG
- TGACGGCCGT CGTAGGGTTG AGAAAGAGTA GAGAGAAGAA GAGAAAGGTC TTAACGACCG AAAAGAAAGC

+ CTGTTCTGCA AATTTCCCGA AACTTCAGAG ACCTAAAACC CCCCAACGAA GGTAAACAAG TAACAACGGA
- GACAAGACGT TTAAAGGGCT TTGAAGTCTC TGGATTTTGG GGGGTTGCTT CCATTTGTTC ATTGTTGCCT

+ AAACCCAGTG TAATTAATTT ATGTATGGTC TTCCTCCTCC TCCCCCTTCT TCTTCTTCTT ATGGTAGATA
- TTTGGGTCAC ATTAATTAAA TACATACCAG AAGGAGGAGG AGGGGGAAGA AGAAGAAGAA TACCATCTAT

+ CTGTAGGCTT GTTGGACAGT TGATTGTGGT GGGTTTTTCT GGGGTTTGTT TTTCTTTATA GATCTGAGAT
- GACATCCGAA CAACCTGTCA ACTAACACCA CCCAAAAAGA CCCCAAACAA AAAGAAATAT CTAGACTCTA

+ GCTAGGGTTT GAGGGCAATA ATCTCTTATT TAGATCTGAG ATTCTAGGGT TTGAGGTCAA GAATCTTTAA
- CGATCCCAAA CTCCCGTTAT TAGAGAATAA ATCTAGACTC TAAGATCCCA AACTCCAGTT CTTAGAAATT

+ AATTATAAAT AAAAAAAAAT ATATGCTAGT TTTTGAGTTT GTGGACGATT ATATATATGT GAAAAGTGGT
- TTAATATTTA TTTTTTTTTA TATACGATCA AAAACTCAAA CACCTGCTAA TATATATACA CTTTTCACCA

+ TGTTTTATTT TATTTATTTG AATTAATTGT TTTTGCAGGG GTAGCAAGAA ATGGTGGAAA GGATGAAAGA
- ACAAAATAAA ATAAATAAAC TTAATTAACA AAAACGTCCC CATCGTTCTT TACCACCTTT CCTACTTTCT

+ GAAGATAAAG ATCAGGAGGA
- CTTCTATTTC TAGTCCTCCT
```

+ 5UTR Py-rich stretch
+ AAGAA-motif
+ Box 4

Site Name	Organism	Position	Strand	Matrix score.	sequence	function
Box 4	Petroselinum crispum	11	+	6	ATTAAT	part of a conserved DNA module involved in light responsiveness
Box 4	Petroselinum crispum	643	+	6	ATTAAT	part of a conserved DNA module involved in light responsiveness
Box 4	Petroselinum crispum	463	+	6	ATTAAT	part of a conserved DNA module involved in light responsiveness
Box 4	Petroselinum crispum	932	+	6	ATTAAT	part of a conserved DNA module involved in light responsiveness

+ Box-W1

Site Name	Organism	Position	Strand	Matrix score.	sequence	function
Box-W1	Petroselinum crispum	825	-	6	TTGACC	fungal elicitor responsive element

+ CAAT-box
+ CATT-motif

Site Name	Organism	Position	Strand	Matrix score.	sequence	function
CATT-motif	Zea mays	90	-	6	GCATTC	part of a light responsive element
CATT-motif	Zea mays	324	+	6	GCATTC	part of a light responsive element

+ CCAAT-box

Site Name	Organism	Position	Strand	Matrix score.	sequence	function
CCAAT-box	Hordeum vulgare	239	+	6	CAACGG	MYBHv1 binding site
CCAAT-box	Hordeum vulgare	624	+	6	CAACGG	MYBHv1 binding site

+ G-box

Site Name	Organism	Position	Strand	Matrix score.	sequence	function
G-box	Zea mays	315	-	6	CACGAC	cis-acting regulatory element involved in light responsiveness

+ GA-motif

Site Name	Organism	Position	Strand	Matrix score.	sequence	function
GA-motif	Glycine max	427	+	8	AAGGAAGA	part of a light responsive element

+ GAG-motif

Site Name	Organism	Position	Strand	Matrix score.	sequence	function
GAG-motif	Spinacia oleracea	518	-	7	AGAGATG	part of a light responsive element

+ GC-motif

Site Name	Organism	Position	Strand	Matrix score.	sequence	function
GC-motif	Zea mays	109	-	6	CCCCCG	enhancer-like element involved in anoxic specific inducibility

+ GCN4_motif

Site Name	Organism	Position	Strand	Matrix score.	sequence	function
GCN4_motif	Oryza sativa	331	-	7	TGAGTCA	cis-regulatory element involved in endosperm expression

+ HSE

Site Name	Organism	Position	Strand	Matrix score.	sequence	function
HSE	Brassica oleracea	383	+	9	AAAAAATTTC	cis-acting element involved in heat stress responsiveness

+ LTR

Site Name	Organism	Position	Strand	Matrix score.	sequence	function
LTR	Hordeum vulgare	577	+	6	CCGAAA	cis-acting element involved in low-temperature responsiveness

+ MBS

Site Name	Organism	Position	Strand	Matrix score.	sequence	function
MBS	Arabidopsis thaliana	344	+	6	CAACTG	MYB binding site involved in drought-inducibility
MBS	Arabidopsis thaliana	717	-	6	CAACTG	MYB binding site involved in drought-inducibility

+ Skn-1_motif

Site Name	Organism	Position	Strand	Matrix score.	sequence	function
Skn-1_motif	Oryza sativa	330	-	5	GTCAT	cis-acting regulatory element required for endosperm expression

+ Sp1

Site Name	Organism	Position	Strand	Matrix score.	sequence	function
Sp1	Zea mays	599	+	5	CC(G/A)CCC	light responsive element
Sp1	Zea mays	669	+	5	CC(G/A)CCC	light responsive element

+ TATA-box
+ TATC-box

Site Name	Organism	Position	Strand	Matrix score.	sequence	function
TATC-box	Oryza sativa	198	-	7	TATCCCA	cis-acting element involved in gibberellin-responsiveness

+ TC-rich repeats

Site Name	Organism	Position	Strand	Matrix score.	sequence	function
TC-rich repeats	Nicotiana tabacum	229	-	9	ATTTTCTTCA	cis-acting element involved in defense and stress responsiveness

+ TCA-element

Site Name	Organism	Position	Strand	Matrix score.	sequence	function
TCA-element	Brassica oleracea	524	-	9	GAGAAGAATA	cis-acting element involved in salicylic acid responsiveness

+ Unnamed__4
+ W box
+ chs-Unit 1 m1

Site Name	Organism	Position	Strand	Matrix score.	sequence	function
chs-Unit 1 m1	Arabidopsis thaliana	244	-	10	ACCTACCACAC	part of a light responsive element

Figure S4

```
+ AATAGTGAGT CTGAGAGAGA TCGAGTAGTT GAAGAGAGGG GGGGTGGGGG GCAGTGGAAG TCAAATCCAT
- TTATCACTCA GACTCTCTCT AGCTCATCAA CTTCTCTCCC CCCCACCCCC CGTCACCTTC AGTTTAGGTA

+ CAGGTGTGGC CGACTCCACG TGAACTCGGA TAAACACCGT ACCGTATAAC TGGAAAACCG GTTCGGTTTT
- GTCCACACCG GCTGAGGTGC ACTTGAGCCT ATTTGTGGCA TGGCATATTG ACCTTTTGGC CAAGCCAAAA

+ GCACCGTACC AAAGTAAATT AAGAAAAAAA AAACAAACCA AACCGATCAG TTATTTTCCG GTTCGATACT
- CGTGGCATGG TTTCATTTAA TTCTTTTTTT TTTGTTTGGT TTGGCTAGTC AATAAAAGGC CAAGCTATGA

+ ATATCTCCAT TCCCTCCACC GCCCCATTTC CTTTCCTTCT ATCAGCCACC TGTTACGTGT CAACTGCACA
- TATAGAGGTA AGGGAGGTGG CGGGGTAAAG GAAAGGAAGA TAGTCGGTGG ACAATGCACA GTTGACGTGT

+ AGCATCGACT GAGGAAGAAG CAGCTTTAGC AACTAGCAAG TGATGCTAGC ATCTCTCAAA AAAGTGGGAC
- TCGTAGCTGA CTCCTTCTTC GTCGAAATCG TTGATCGTTC ACTACGATCG TAGAGAGTTT TTTCACCCTG

+ ACGTGAAAAG AAATAGGCTA AACCGACAAT AAACGGTGGG ATAGCATTCA TCACACCAAC CGCATCGATC
- TGCACTTTTC TTTATCCGAT TTGGCTGTTA TTTGCCACCC TATCGTAAGT AGTGTGGTTG GCGTAGCTAG

+ ACCGTTGATT ATCGTTCCAT GTTTCTTACC TTATTTGCCC AACAAAATAT CCAAAATATC TAAAAATAGA
- TGGCAACTAA TAGCAAGGTA CAAAGAATGG AATAAACGGG TTGTTTTATA GGTTTTATAG ATTTTTATCT

+ AAAAGCAACA AACAGCGGAT ATTAATAAAA GATTTATCCA AGGTCTTCCA TATATGGGAA AATTCATTTA
- TTTTCGTTGT TTGTCGCCTA TAATTATTTT CTAAATAGGT TCCAGAAGGT ATATACCCTT TTAAGTAAAT

+ GCGACCTTTC CTTTCTTGAA CAGCCTGCAG CACCAACTCT CTCTCTCTCC TCAGTCTTCT CCTCTTTCCA
- CGCTGGAAAG GAAAGAACTT GTCGGACGTC GTGGTTGAGA GAGAGAGAGG AGTCAGAAGA GGAGAAAGGT

+ AAATTGCTCC GAAAATTTCC AGTTCAGACC TAAACTTCAG ACCTAAGACC CCTCAACGAA GGTAAACAAG
- TTTAACGAGG CTTTTAAAGG TCAAGTCTGG ATTTGAAGTC TGGATTCTGG GGAGTTGCTT CCATTTGTTC

+ AGAACCAGAG GTGAACCCAG TACTTAGTTT ATCTATGTTT TTCCTTCTTT TTTTTTCTTT TTTCCTTCTA
- TCTTGGTCTC CACTTGGGTC ATGAATCAAA TAGATACAAA AAGGAAGAAA AAAAAAGAAA AAAGGAAGAT

+ TTAATGGTGA ATACTAAAAA CTCTAGGCTT GTCTGGCAGT TGATTGTGGT TGGTGGGTTG ATCTGTGTTT
- AATTACCACT TATGATTTTT GAGATCCGAA CAGACCGTCA ACTAACACCA ACCACCCAAC TAGACACAAA

+ TATTTTACTT ATTTAGATCT GAGATATGTT AGGGTATGAG GTCAATAATC TTAAAATTAT CAGAAACCCA
- ATAAAATGAA TAAATCTAGA CTCTATACAA TCCCATACTC CAGTTATTAG AATTTTAATA GTCTTTGGGT

+ GAAAATATAC TGTAGTTTGT GAAACATTTA TATATATATG TGGAAATTTA AAGTGATTGG TTTATTTTAT
- CTTTTATATG ACATCAAACA CTTTGTAAAT ATATATATAC ACCTTTAAAT TTCACTAACC AAATAAAATA

+ TTTATTTGAA TTATTGTTTT
- AAATAAACTT AATAACAAAA
```

+ 4cl-CMA2b

Site Name	Organism	Position	Strand	Matrix score.	sequence	function
4cl-CMA2b	Petroselinum crispum	401	+	10	TCTCACCAACC	light responsive element

+ AAGAA-motif
+ ABRE

Site Name	Organism	Position	Strand	Matrix score.	sequence	function
ABRE	Arabidopsis thaliana	87	+	6	CACGTG	cis-acting element involved in the abscisic acid responsiveness
ABRE	Arabidopsis thaliana	350	+	6	CACGTG	cis-acting element involved in the abscisic acid responsiveness
ABRE	Arabidopsis thaliana	264	+	6	TACGTG	cis-acting element involved in the abscisic acid responsiveness

+ AC-I
+ ACE

Site Name	Organism	Position	Strand	Matrix score.	sequence	function
ACE	Petroselinum hortense	85	-	7	ACGTGGA	cis-acting element involved in light responsiveness

+ AE-box

Site Name	Organism	Position	Strand	Matrix score.	sequence	function
AE-box	Arabidopsis thaliana	439	-	8	AGAAACAT	part of a module for light response

+ ARE

Site Name	Organism	Position	Strand	Matrix score.	sequence	function
ARE	Zea mays	175	-	6	TGGTTT	cis-acting regulatory element essential for the anaerobic induction
ARE	Zea mays	968	+	6	TGGTTT	cis-acting regulatory element essential for the anaerobic induction

+ Box 4

Site Name	Organism	Position	Strand	Matrix score.	sequence	function
Box 4	Petroselinum crispum	511	+	6	ATTAAT	part of a conserved DNA module involved in light responsiveness
Box 4	Petroselinum crispum	770	+	6	ATTAAT	part of a conserved DNA module involved in light responsiveness

+ Box-W1

Site Name	Organism	Position	Strand	Matrix score.	sequence	function
Box-W1	Petroselinum crispum	880	-	6	TTGACC	fungal elicitor responsive element

+ C-repeat/DRE

Site Name	Organism	Position	Strand	Matrix score.	sequence	function
C-repeat/DRE	Arabidopsis thaliana	77	+	8	TGGCCGAC	regulatory element involved in cold- and dehydration-responsiveness

+ CAAT-box
+ CATT-motif

Site Name	Organism	Position	Strand	Matrix score.	sequence	function
CATT-motif	Zea mays	394	+	6	GCATTC	part of a light responsive element

+ CCAAT-box

Site Name	Organism	Position	Strand	Matrix score.	sequence	function
CCAAT-box	Hordeum vulgare	422	-	6	CAACGG	MYBHv1 binding site

+ G-Box

Site Name	Organism	Position	Strand	Matrix score.	sequence	function
G-Box	Pisum sativum	87	+	6	CACGTG	cis-acting regulatory element involved in light responsiveness
G-Box	Pisum sativum	350	+	6	CACGTG	cis-acting regulatory element involved in light responsiveness
G-Box	Antirrhinum majus	264	-	6	CACGTA	cis-acting regulatory element involved in light responsiveness

+ G-box

Site Name	Organism	Position	Strand	Matrix score.	sequence	function
G-box	Brassica napus	86	-	7	CACGTGG	cis-acting regulatory element involved in light responsiveness
G-box	Daucus carota	264	+	6	TACGTG	cis-acting regulatory element involved in light responsiveness
G-box	Arabidopsis thaliana	87	+	6	CACGTG	cis-acting regulatory element involved in light responsiveness
G-box	Arabidopsis thaliana	350	+	6	CACGTG	cis-acting regulatory element involved in light responsiveness

+ GA-motif

Site Name	Organism	Position	Strand	Matrix score.	sequence	function
GA-motif	Arabidopsis thaliana	729	-	8	ATAGATAA	part of a light responsive element

+ GAG-motif

Site Name	Organism	Position	Strand	Matrix score.	sequence	function
GAG-motif	Spinacia oleracea	330	-	7	AGAGATG	part of a light responsive element
GAG-motif	Arabidopsis thaliana	596	-	7	AGAGAGT	part of a light responsive element

+ GT1-motif

Site Name	Organism	Position	Strand	Matrix score.	sequence	function
GT1-motif	Solanum tuberosum	813	+	10	ATGGTGGTTGG	light responsive element

+ I-box

Site Name	Organism	Position	Strand	Matrix score.	sequence	function
I-box	Arabidopsis thaliana	427	-	9	acGATAATC	part of a light responsive element

+ L-box

Site Name	Organism	Position	Strand	Matrix score.	sequence	function
L-box	Petroselinum crispum	401	+	10	TCTCACCAACC	part of a light responsive element

+ LTR

Site Name	Organism	Position	Strand	Matrix score.	sequence	function
LTR	Hordeum vulgare	639	+	6	CCGAAA	cis-acting element involved in low-temperature responsiveness

+ MBS

Site Name	Organism	Position	Strand	Matrix score.	sequence	function
MBS	Arabidopsis thaliana	117	+	6	TAACTG	MYB binding site involved in drought-inducibility
MBS	Arabidopsis thaliana	271	+	6	CAACTG	MYB binding site involved in drought-inducibility
MBS	Arabidopsis thaliana	188	-	6	TAACTG	MYB binding site involved in drought-inducibility
MBS	Arabidopsis thaliana	807	-	6	CAACTG	MYB binding site involved in drought-inducibility

+ Sp1

Site Name	Organism	Position	Strand	Matrix score.	sequence	function
Sp1	Zea mays	38	-	5	CC(G/A)CCC	light responsive element
Sp1	Oryza sativa	229	-	6	GGGCGG	light responsive element
Sp1	Zea mays	42	-	5.5	CC(G/A)CCC	light responsive element
Sp1	Zea mays	39	-	5	CC(G/A)CCC	light responsive element
Sp1	Zea mays	46	-	5	CC(G/A)CCC	light responsive element

+ TATA-box
+ TATC-box

Site Name	Organism	Position	Strand	Matrix score.	sequence	function
TATC-box	Oryza sativa	387	-	7	TATCCCA	cis-acting element involved in gibberellin-responsiveness

+ TCA-element

Site Name	Organism	Position	Strand	Matrix score.	sequence	function
TCA-element	Nicotiana tabacum	743	+	9	CCATCTTTTT	cis-acting element involved in salicylic acid responsiveness

+ TCT-motif

Site Name	Organism	Position	Strand	Matrix score.	sequence	function
TCT-motif	Arabidopsis thaliana	444	+	6	TCTTAC	part of a light responsive element

+ Unnamed__1
+ Unnamed__11
+ Unnamed__3
+ Unnamed__4
+ W box
+ box S
+ circadian

Site Name	Organism	Position	Strand	Matrix score.	sequence	function
circadian	Lycopersicon esculentum	62	+	6	CAANNNNATC	cis-acting regulatory element involved in circadian control
circadian	Lycopersicon esculentum	407	+	6	CAANNNNATC	cis-acting regulatory element involved in circadian control
circadian	Lycopersicon esculentum	179	+	6	CAANNNNATC	cis-acting regulatory element involved in circadian control

Figure S5

B-type (upstream of *PpDAM2*=*ProDAM2*):

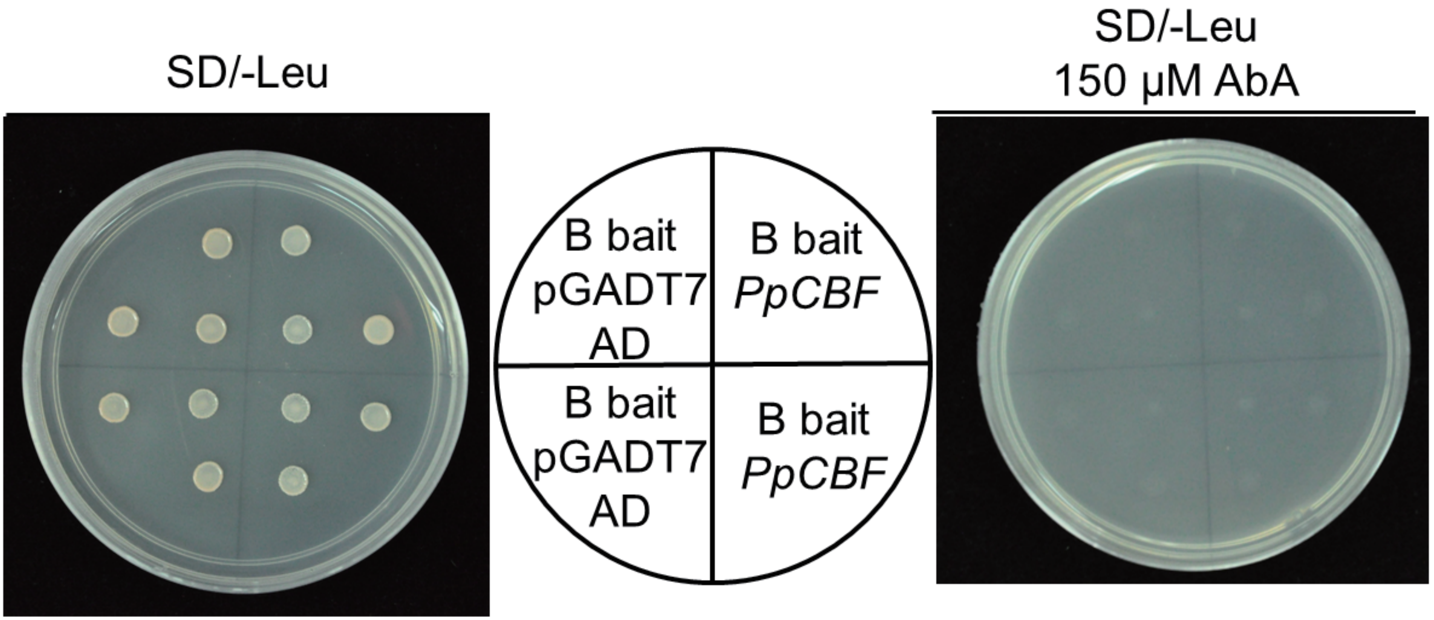

Figure S6
